# Realist theory construction for a mixed method multilevel study of neighbourhood context and postnatal depression

**DOI:** 10.1186/s40064-016-2729-9

**Published:** 2016-07-15

**Authors:** John G. Eastwood, Lynn A. Kemp, Bin B. Jalaludin

**Affiliations:** Community Paediatrics, Sydney Local Health District, Croydon Community Health Centre, 24 Liverpool Road, Croydon, NSW 2132 Australia; School of Public Health and Community Medicine, The University of New South Wales, Sydney, NSW 2052 Australia; School of Women’s and Children’s Health, The University of New South Wales, Sydney, NSW 2052 Australia; Ingham Institute of Applied Medicine, The University of New South Wales, Liverpool, NSW 2170 Australia; School of Public Health, The University of Sydney, Sydney, NSW 2006 Australia; School of Medicine, Griffith University, Gold Coast, QLD 4222 Australia; School of Nursing and Midwifery, Western Sydney University, Campbelltown, NSW 2560 Australia

## Abstract

**Background:**

We have recently described a protocol for a study that aims to build a theory of neighbourhood context and postnatal depression. That protocol proposed a critical realist *Explanatory Theory Building Method* comprising of an: (1) emergent phase, (2) construction phase, and (3) confirmatory phase. A concurrent triangulated mixed method multilevel cross-sectional study design was described. The protocol also described in detail the *Theory Construction Phase* which will be presented here.

**Methods:**

The *Theory Construction Phase* will include: (1) defining stratified levels; (2) analytic resolution; (3) abductive reasoning; (4) comparative analysis (triangulation); (5) retroduction; (6) postulate and proposition development; (7) comparison and assessment of theories; and (8) conceptual frameworks and model development.

**Theory construction:**

The stratified levels of analysis in this study were predominantly social and psychological. The abductive analysis used the theoretical frames of: Stress Process; Social Isolation; Social Exclusion; Social Services; Social Capital, Acculturation Theory and Global-economic level mechanisms. Realist propositions are presented for each analysis of triangulated data. *Inference to best explanation* is used to assess and compare theories. A conceptual framework of maternal depression, stress and context is presented that includes examples of mechanisms at psychological, social, cultural and global-economic levels. Stress was identified as a necessary mechanism that has the tendency to cause several outcomes including depression, anxiety, and health harming behaviours. The conceptual framework subsequently included conditional mechanisms identified through the retroduction including the stressors of isolation and expectations and buffers of social support and trust.

**Conclusion:**

The meta-theory of critical realism is used here to generate and construct social epidemiological theory using stratified ontology and both abductive and retroductive analysis. The findings will be applied to the development of a middle range theory and subsequent programme theory for local perinatal child and family interventions.

**Electronic supplementary material:**

The online version of this article (doi:10.1186/s40064-016-2729-9) contains supplementary material, which is available to authorized users.

## Background

It is increasingly recognised that maternal and paternal depression has a significant impact on the developmental trajectory of infants and children both before and after birth. Much is now known about the genetic, biological, physiological and clinical influences on the genesis of depression (Gluckman and Hanson 2006; Matthews and Meaney 2005; Meaney 2010; Osborne and Monk 2013; Monk et al. 2012; Meltzer-Brody 2011) and its subsequent impact on the unborn foetus, infants and other family members (Beck 1995; Murray et al. 1996; Martins and Gaffan 2000).

We have previously reported on individual level psychosocial predictors of postnatal depression in South Western Sydney and proposed that “the findings were consistent with group-level socioeconomic deprivation, neighbourhood environment, social networks and ethnic diversity having causal effects on postnatal depressive symptomatology and other perinatal outcomes” (Eastwood et al. 2011). We observed that the proposition was consistent with a recent qualitative study by O’Campo and colleagues “Neighbourhoods and mental well-being” (O’Campo et al. 2009) which found that “neighbourhood affordability, negative community factors including crime and vandalism, and social makeup including unemployment and poverty, were felt to be associated with poor mental wellbeing” (Eastwood et al. 2013).

For the purposes of designing a theory driven population-based intervention programme we have undertaken to build a theory of maternal depression, the developmental origins of health and disease, and neighbourhood context. Drawing on recent criticism of social epidemiological studies and multi-level studies in particular (Muntaner 1999; Krieger 2001; O’Campo 2003; Carpiano and Daley 2006; Raphael 2006) we have used a realist explanatory theory building method (Eastwood et al. 2014).

The purpose of this manuscript is to report on the findings of the Construction Phase of the main study (Eastwood 2011).

## Methods

### Introduction

The Commonwealth of Australia has six States and two territories. Sydney is the capital city of the State of New South Wales (NSW) on the east coast. The study area is four local government city councils in South Western Sydney; has a diverse multicultural population with 28.4 % of the population born overseas compared with 17.8 % for the rest of NSW; and is an area of substantial social disadvantage, and have lower education attainment and lower income levels then other parts of NSW.

**Fig. 1:**
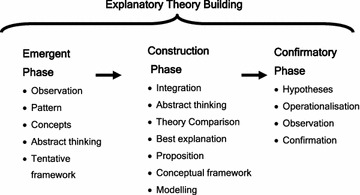
Phases of Explanatory Theory Building

Critical realism provided the methodological underpinning for this mixed method multilevel study. Critical realism assumes ontological and hierarchical stratification of reality (Danermark 2002) making it suitable for the examination of social and psychosocial phenomenon such as socio-economic stratification, social exclusion, isolation and cultural context. For critical realists, causation is not solely based on observed regularities in data (i.e., correlations or regression studies) but also on identifying the underlying causal mechanisms and how they work. We have previously reported on the protocol for the main study which used both *Emergent* and *ConstructionPhases* of a realist *Explanatory Theory Building Method* (Fig. 1) (Eastwood et al. 2014).

The concurrent triangulation design used in this study is one of the most commonly used mixed method designs (Tashakkori and Teddlie 2003, p. 229). The research design used for this study sought to achieve the standards of integration proposed by Yin (2006) with integration occurring through use of common research questions, study design, units of analysis, samples for study and analytic strategies during both the *Emergent* and *Construction* phases.

During the *Emergent Phase* a constant comparative approach between qualitative (intensive) and quantitative (extensive) study arms was used. The methods used to detect and describe the phenomena under study included: interviews, focus groups, exploratory data analysis, exploratory factor analysis, regression, and exploratory ecological and multilevel spatial data analysis (Eastwood et al. 2012a, b, 2013a, b, c, 2014b). Theory generation was undertaken in both the qualitative and quantitative arms of the Emergent Phase, to develop tentative conceptual frameworks. This analysis used: categorical principal component analysis, exploratory factor analysis, exploratory confirmatory factor analysis, coding of concepts and categories, constant comparative analysis, drawing of conceptual networks, and situational analysis, to move from the “concrete to the abstract” (Danermark et al. 2002, p. 109).

### Construction phase methods

The purpose of the *Theory Construction Phase* is to undertake abductive triangulation of the findings from the mixed method studies conducted in the *Emergent Phase* in order to construct a conceptual framework, theory and model. We have previously reported on the *Construction Phase* methods which will be summarised below (Eastwood et al. 2014). The methods include: (1) defining stratified levels; (2) analytic resolution; (3) abductive reasoning; (4) comparative analysis (triangulation); (5) retroduction; (6) postulate and proposition development; (7) comparison and assessment of theories; and (8) conceptual frameworks and model development.

#### Stratified levels

A hallmark of critical realist analysis is the ontological assumption that reality consists of hierarchically ordered levels where a lower level creates the conditions for a higher level. The higher level is not, however, determined by the lower level and has its own “generative mechanisms”. The above approach is useful as an analytical framework but “in reality levels are entwined and [the] mechanisms could be supporting each other or counteracting each other, and the outcome in a specific context is the result of a very complex interplay between levels and mechanisms” (Danermark and Gellerstedt 2004, p. 350). In this study we will focus the analysis on mechanisms operating at the psychosocial and social levels while maintaining awareness of the existence and importance of the other levels.

#### Analytic resolution

Analytic resolution was undertaken in both the *Emergent* qualitative and quantitative studies as part of procedures such as coding of categories, situational analysis and factor analysis. Further analytic resolution was also undertaken during the *Theory Construction Phase*. The process contributes to the identification of the best theoretical explanation. A risk of this process is the loss of detail regarding the complexity of the processes under study. To partially address this, the analytic process involves checking back to the empirical findings in both the qualitative and quantitative studies.

#### Abductive reasoning

Abductive reasoning is the hallmark of realist reasoning. It is the reinterpretation and recontextualisation of phenomena within a conceptual framework or a set of ideas. Modell (2009, p. 213) observes that “abduction does not move directly from empirical observations to theoretical inferences, as is the case in purely inductive research, but relies heavily on theories as mediators for deriving explanations”. We approached the abductive process in three stages. The first step was to recontexualise, or redescribe the phenomena identified within one of the more abstract concepts emerging from the *Emergent Phase,* such as social capital or “big business”. This abductive inference was imbedded within the theory generation processes of the *Emergent Phase*. The second stage, reported here. recontexualises the observed phenomena through the lens of theories arising from literature, key informants and the earlier theory generation (such as social capital or acculturation theory). Finally abduction will be undertaken as part of the *Inference to best Explanation,* as reported here.

#### Comparative analysis (triangulation)

As discussed above we will use an integration of methods, data collection and analysis as proposed by Yin (2006) and Woolley (2009). Comparative analysis was used during the *Emergent Phase* and in this way the two arms of the study remained integrated. In the *Theory Construction Phase* findings from the intensive (qualitative) and extensive (quantitative) study arms are compared. The intensive qualitative studies provide causal explanations of possible mechanisms while the extensive quantitative studies assist with distinguishing regularities, patterns and features of the population groups. During this phase of the comparative analysis the relevant literature was reviewed in more depth and treated as a third source of information for the comparative analysis. Divergence of findings was given particular attention as it is here that “new” knowledge or understanding can be elicited through the abductive and retroductive reasoning.

#### Retroduction

Retroduction is a process where we move from a description and analysis of concrete phenomena to reconstruct the basic conditions for those phenomena to be what they are. In this way thought operations and counterfactual thinking are used to argue toward counterfactual and transfactual conditions. In critical realist analysis of causal inference structures, or entities, are viewed as having causal powers, or mechanisms, that have a tendency to produce events or outcomes. Contextual conditions thus play an important role in the realist understanding of causality because causal powers may only result in an event occurring under certain conditions (Pawson 2006).

#### Postulates and propositions development

**Fig. 2:**
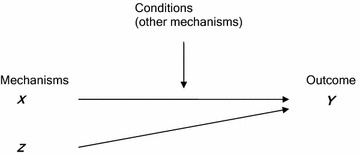
Graphical representation of critical realist propositions (Danermark 2002)

From a hypothetico-deductive perspective Dubin (1969, p. 205) states that propositions are often expressed as “if *a* then *b*” deductive statements. The hypothetico-deductive approach to theory building requires a “closed” system and that theory be tested within the empirical world with “things observable” (Dubin 1969, p. 205). By contrast critical realism views reality as an “open system” where causative processes are always contextually determined. Critical realism also seeks to discover the hidden mechanisms that explain the empirical phenomena. Thus realist theoretical propositions are about how “mechanisms (M) are fired in contexts (C) to produce outcomes (O)” (Pawson and Tiley 1997, p. 85). Causal relationships only occur when the generative mechanism comes into operation. Sometimes different mechanisms produce the same outcome. The contextual conditions determine whether the generative mechanism(s) will come into play and the nature of the outcome. The contextual conditions include other mechanisms that may either trigger or counteract the causal mechanism. A graphical representation critical realist propositions will be used here (Fig. 2) to summarise the findings of the comparative analysis (triangulation), and abductive and retroductive reasoning.

#### Comparison and assessment of theories

Critical realist methodologist Danermark et al. (2002), and Haig (2005) both identify a stage in explanatory research and theory construction where comparison and assessment of the identified theories and abstractions is undertaken. Haig (2005) argued for Thagard’s (1992) formulation of Inference to the Best Explanation, which included the seven principles of: symmetry, explanation, analogy, data priority, contradiction, competition, and acceptability, and three criteria: consilience, simplicity, and analogy (Thagard 1988). Ward (2009) argued that the commonly used Bradford Hill “criteria” are an Inference to Best Explanation within a realist epistemology. We will, therefore, use here both the Thagard and Hill principles of best explanation as part of the abductive process and assessment of theories.

#### Conceptual frameworks and models

Finally we will use the typology of conceptual frameworks, theory and models proposed by Carpiano and Daley (2006) where the different levels of abstraction move from the broadest level of conceptualisation (framework) to the more focused (model). A model may draw on several theories and when presented as a diagram a conceptual model may provide a visualisation of proposed causal linkages.

## Theory construction (results and discussion)

### Emergent theory

As described above the *Emergent Phase* used a constant comparative approach between qualitative (intensive) and quantitative (extensive) study arms. The findings of those studies have been previously reported (Eastwood et al. 2012a, b, 2013a, b, c, 2014b). Theory generation was undertaken in both the qualitative and quantitative arms of the Emergent Phase, to develop a tentative conceptual framework.

Stress emerged as a cause of both antenatal and postnatal depression with causes including: loss of control, lost expectation, isolation, economic hardship, marginalisation and infant temperament. The literature reviews identified empirical studies that suggested that antenatal stress and depression resulted in altered infant temperament which in turn was a cause of postnatal stress and depression. Other empirical studies indicated that antenatal maternal stress had effects on the foetus that potentially affected the life course. Similarly there were studies indicating a causal link between postnatal depression, poor maternal-infant attachment and detrimental impacts on child development. Support emerged as a protective factor against social marginalisation, isolation, stress and loss of control. That support was in turn influenced by family, social networks or social capital, social cohesion and social services. The quantitative studies supported the above emerging causal network but also indicated that ethnic diversity played a complex role in relation to social support. The macro role of globalisation was identified as impacting on economic and social marginalisation and maternal expectations.

The above conceptual framework identifies structures and mechanisms operating at different levels. Examples of possible structures, mechanisms, contexts and outcomes are shown in Table 1, based on the findings from the *Emergent Theory Building.*

**Table 1:** Analytical Levels of Depression and Context

Levels	Example of structures	Example of mechanisms	Example of contexts	Example of negative outcomes
Global economic	Multinational companies	Exploitation, Profit	Labour market	Unemployment, migration
Cultural	Ethnic community	Segregation	Migration	Bonding networks
Social	Neighbourhood social capital	Relationships	Social networks	Isolation
Social	Family	Emotional support	Absent partner	Isolation
Psychological	Self	Relation to self	Isolation	Feeling overwhelmed and alone
Psychological	Mind	Stress	Overwhelmed and alone	Depression, reduced motivation, anxiety
Biological	Body	Neurobiological	Reduced motivation	Hypoactivity of motivation areas

Abductive reasoning is central to the critical realist explanatory method. One approach to abductive reasoning is to recontexualise or redescribe the phenomenon within a number of frames of interpretation or theories. The theoretical and conceptual frameworks selected for the analysis were chosen after considering a wide range of relevant social epidemiological, social, community psychology, public health, perinatal and postnatal depression theories including: social isolation and social exclusion theories, the stress process model (Pearlin 1989), social network and social capital theories, theories of globalisation, psychosocial and social support theories, life-course theory, eco-social theory (Krieger 2001), materialist and neo-material theories, transactional theory, the ecological model for development (Bronfenbrenner 1979), acculturation theory, integrated perinatal models, and theoretical models of neighbourhood effects (Green and Ottoson 1999; Wandersman and Nation 1998; Ellen et al. 2001).

For the purposes of this manuscript we have limited the analysis to explaining causal mechanisms at the social level in relation to the following theoretical frames:

1. Stress Process Model
2. Social Isolation Theory
3. Social Exclusion Theory
4. Social Services
5. Social Capital Theory
6. Global-Economic Level Mechanisms.

### Stress process theory

#### Introduction

Stress was identified as a phenomenon causing depression in both the qualitative studies and literature review (Eastwood 2011; Eastwood et al. 2015). The stress process model was first described by Pearlin et al. (1981). The model is concerned with explaining ways in which social structure influence mental health with a focus on the connection between disadvantaged social status and psychopathology. Redescription and recontextualisation of perinatal stress, and depression, will be undertaken here within the conceptual framework provided by the “Stress Process Model”. The analysis is intentionally limited to the social levels and will not consider our findings regarding the role of infant temperament (Eastwood et al. 2012a, b, 2015).

According to the stress theory “stressors occur either because of psychological characteristics of individuals or because of environmental factors over which the person has little control. The effects of these stressors may be to increase psychopathology, increase psychological growth, or to lead to no permanent change in the individual” (Sandler et al. 2000). Baron and Kenny (1986) proposed that these “stressful experiences can be viewed from a causal perspective with different types of stress affecting each other in complex ways to lead to the development of psychological and physical health problems”. Stress Theory is considered particularly applicable to the development of a theoretical model of postnatal depression and will be discussed further below.

#### Triangulation

The qualitative arm of the study identified stress as a cause of both antenatal and postnatal depression (Eastwood 2011, 2015). While the term “stress” was only mentioned by one mother, we interpreted the coded category “loss of control” as expressing a similar concept. The phase two focused coding related a number of sub-codes to the concept “Stress” and it subsequently became a “core” category for the qualitative analysis. The Individual Level Qualitative Theory Generation confirmed stress to be a strong contender as the proximal cause of depression. The quantitative studies were not able to directly measure stress as a variable.

In the Individual Level Quantitative Theory Generation we abductively inferred that the latent variables F2 and F4 in the non-linear principal component analysis may be representative of exclusion or marginalisation respectively (Eastwood et al. 2012). The loading of poor self-reported health on those latent variables gave support to the idea that this may represent stress or something similar.

Previous research has identified stressful events as potentially having an effect on postnatal depression (marital relationship, housing, finances, unemployment, bereavement), and that the influence such stressors is related to the woman’s perception and appraisal of the event (Stein et al. 1989; O’Hara 1995; Lane et al. 1997; Zelkowitz and Milet 1995). The NHMRC Systematic Review (NHMRC 2000) found evidence “linking postnatal depression with significant stresses and increased recent negative life events”. Recent reviews of research at the neurobiological and psychological levels support the role that stress plays in the genesis of depression (Stone et al. 2008; Kinderman 2005).

The stress process can be summarised as follows: “an individuals’ location in the social structure has an associated inequality in resources, status, and power that differentially expose them to stressors which can damage their physical and/or psychological health. The damage may be moderated or lessened by their psychological resources and coping strategies which are socially patterned in ways that can leave members of disadvantaged groups more vulnerable to the harmful effects of stress” (Pearlin 1989).

Two pathways that may link social structure with stress are proposed to be “exclusion from full participation in the social system and participation that fails to provide the expected returns” (Aneshensel 1992). Socio-economic class, ethnicity, race, gender and age may result in the unequal distribution of opportunities and resources. Thus low social status may itself be a source of stress (Pearlin 1989).

Aneshensel (1992) argues that the “structural perspective on social causation understands stress both as a consequence of location in the social system and as a determinant of some outcome, most typically psychological distress. … Location in the social system influences the risk of encountering stressors, which in turn influences the chances of becoming emotionally distressed”. Hinkel (1987) also notes that “societies prescribe a variety of forms of behaviour, conditions and relationships that are proper for its members with sanctions for stepping outside these prescriptions acting as a form of stress”. This may have implications for certain ethnic and cultural groups.

Three types of moderators of the stress process are coping strategies, personal resources and social support. Coping strategies are behavioural or psychological state changes that people can make themselves. To make these coping strategies individuals need personal resources which may be either personal or social. Personal resources include notions of is self-esteem and self-mastery. Social support has been consistently shown to have a strong buffering effect on stress outcomes. Social support literature is discussed in more detail below.

#### Abductive and retroductive analysis

Recontexualisation of perinatal depression within a Stress Process Model provides an explanation of the causes of perinatal depression. External social or environmental and internal biological or psychological stressor mechanisms are able to “trigger” a causative mechanism called *Stress* at the psychological and biological levels that result in the phenomenon of depression. *Stress* is defined here as a “necessary” mechanism.

The conditional “stressors” mechanisms may be *event stressor* such as poor health, childbirth experience, loss of status, loss of financial income, loss of control, unmet expectations, irritable infant and partner behaviour. *Chronic stressors* may also contribute and include: *status strains* (i.e., race, sole parent, poverty, and religion), *role strains* (i.e., wife, motherhood, and daughter), *ambient strains* (i.e., neighbourhood crime, services, physical decay), and *quotidian strains* (i.e., loneliness, daily hassle of sole parenthood). The Stress Process Model is able to explain our observed association of financial stress and infant temperament with depressive symptoms (Eastwood et al. 2012b, 2014b).

Consideration of the transfactual makes it clear that stressor mechanisms may not result in depression if moderator mechanisms are active. Some of those moderators will be the counterfactual of the causative mechanisms identified above. Moderator mechanisms that have been described include coping resources, personal resources and social support. The Stress Process Model is thus able to explain the observed protective effect of practical, emotional and social support on depressive symptoms.

The Stress Process Model also explains that there may be more than one *Stress Outcome.* These may be depression, anxiety, or drug/alcohol abuse. We also consider here that “self-reported poor health” may be an observed variable associated with *Stress Outcome.*

**Fig. 3:**
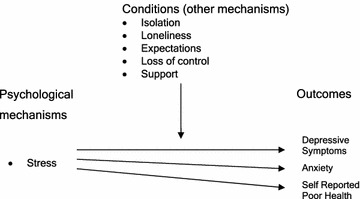
Critical realist model of the stress proposition

A critical realist model of the mechanism stress shown in Fig. 3.

Based on the above analysis the following propositions are made:

- Stress is the mechanism that causes depression when certain personal characteristics and contextual conditions exist
- Stress is positively associated with depression when there is a past medical history of depression, difficult infant temperament including crying, financial stress, loss of expectations, a feeling of “loss of control”, isolation, and lack of support.

Our assessment of *Inference to the Best Explanation* for the above propositions, using Hills “aspects of association” and Thagard’s principles and criteria, is shown (Table 2).

**Table 2:** Stress proposition—inference to best explanation

Criteria	Assessment
*Hill’s aspects of association*	
Strength	Expectation and lack of support both have strong associations with depression
Consistency	The role of stress as a cause of depression has been found in different situations. The role of expectations and lack of support has also been found
Specificity	No specificity identified
Temporality	No temporality demonstrated in this study
Biological gradient	Higher the causes of stress the higher the observed depression
Plausibility	The association between stress and depression is biologically plausible
Coherence	The association is coherent with what is know
Experimental evidence	Interventions that provide support have been demonstrated to reduce depression
Analogy	There is an analogy between the effect of loss of expectation and loss of support. Both result in a similar effect.
*Thagard’s Principles*	
Symmetry	There is symmetry between stress causing depression and support preventing depression
Explanation	The stress proposition a) coheres with evidence on depression, b) evidence on role of support, isolation, loss of control, and c) is single proposition.
Analogy	Stress causing depression is coherent with stress causing anxiety and physiological changes to H-P axis
Data priority	Proposition describes observation re isolation, expectations, support.
Contradiction	There are no contradictory proposals
Competition	No competitive explanation identified where p and q were not explanatorily connected
Acceptance	The stress proposition is coherent with the overall system of propositions
*Thagard’s Criteria*	
Consilience	The central role of stress as an explanation for depression explains the largest range of facts
Simplicity	Stress as a necessary cause of depression is a simple explanation
Analogy	

In summary the stress proposition is an explanation of a “necessary” cause of depression. It explains much of the observed and known evidence but is insufficient on its own. The stress proposition could be used as the basis for building a more complete explanation.

### Social isolation theory

#### Introduction

Isolation and loneliness were identified as phenomenon associated with postnatal depression (Eastwood 2011; Eastwood et al. 2015). We have used abductive logic to redescribe and recontexualise the phenomena of isolation and loneliness associated with postnatal depression within the theoretical concept of social isolation. The central purpose in this section is to ascribe meaning to the phenomenon of postnatal depression within a social isolation conceptual framework.

#### Triangulation

The possibility of social isolation as a “generative” mechanism of the phenomenon of postnatal depression emerged earlier in the exploratory phase. The literature review (Eastwood 2011) and individual level quantitative study (Eastwood et al. 2012a) both provided empirical evidence that lack of social support was a risk factor for postnatal depression. The qualitative study (Eastwood et al. 2015) identified marginalisation and isolation as mechanisms that could result in stress and depression. Examination of the social worlds map drew attention to the situation that may be experienced by most mothers. Buried in the voices of the mothers were stories of “being alone” with the crying infant, with an absent partner, mother or other support friend” (Eastwood 2011).

At the group level social marginalisation and isolation emerged in relation to social support. One of our mothers group drew attention to the role of language and culture noting that “may be some people don’t speak English, we have a special language group … if they don’t know they just isolate…”. This link between isolation and culture we will explore later. Social isolation was also linked to access to transport, financial situation, social networks and “bonding” and “bridging” forms of social capital (Eastwood et al. 2014b).

The group level quantitative studies did not include specific measures of marginalisation and social isolation but did include the counterfactual of social support (i.e., no social support). The ecological studies (linear regression and spatial) found an association of aggregated postnatal depression with apartment living, entropy index, percent of one parent families, nurse visiting rates, smoking rates and aggregated “no social support” (Eastwood et al. 2013a, 2014c). When controlling for individual level factors the multilevel study paradoxically found protective associations with high density, poor social support, and poor schooling. The stratified multilevel studies found that these associations were strongest for mothers not born in Australia (Eastwood et al. 2013c). Abductive analysis (Theory Generation) using the findings from the Factor Analysis suggested that weak bonding networks and high ethnic diversity protected mothers in those communities from depression (Eastwood 2011, pp. 314–323).

Recontextualisation of these multilevel findings within the social isolation conceptual framework suggests that weak bonding networks and high suburb ethnic diversity protect mothers from social isolation. The converse logic is that strong bonding networks in suburbs with low ethnic diversity contribute to social isolation (Eastwood et al. 2013c).

Much of the relevant research on isolation and loneliness has been undertaken through qualitative studies. Beck (2002) undertook a metasynthesis of 18 qualitative studies and identified four overarching themes: (a) incongruity between expectations and the reality of motherhood, (b) spiralling downward, (c) pervasive loss, and (d) making gains. For the theme “spiralling downward” Beck reported the sub-concepts of “anxiety, overwhelmed, obsessive thinking, cognitive impairment, isolation and loneliness, guilt, and contemplating harming oneself” (Beck 2002).

In her metasynthesis Beck (2002) found that mothers soon began a downward spiral of postpartum depression as their feelings worsened. All of the 18 studies included in her metasynthesis included aspects of this downward spiral. The feelings included: depression, sadness, anger, guilt, being overwhelmed, thought of harming themselves, anxiety and loneliness

Beck describes post-partum women as being enveloped in “unbearable loneliness due to the discomfort they felt being around other and their belief that no one else really understood what they were experiencing” (Beck 2002). Beck noted that “as mothers silenced themselves and withdrew socially they felt a profound sense of isolation and loneliness”. Beck (2002) also found that different cultural contexts influenced the degrees of isolation and loneliness. Hmong mothers living in the United States received a high level of family support for a 30 day protected period and did not experience the isolation and loneliness that Jordanian mothers living in Australia suffered (Beck 2002, p. 464).

There have been few quantitative studies that have separately assessed the role of “isolation” or “social isolation”. Birkeland et al. (2005) in their study of adolescent motherhood and postpartum depression identified social isolation, maternal self-efficacy and weight/shape disturbance as being significantly predictive of depression. In their study social isolation had been a subscale of the Parental Stress Index (Abidin 1995).

In a large Danish population-based study (n = 6790) of postnatal depression, the risk factors included “psychological distress in late pregnancy [OR 6.3 (95 % CI 4.4–9.1)], perceived social isolation during pregnancy [OR 3.6 (95 % CI 1.9–7.0)] and a positive history of pre-pregnant psychiatric disease [OR 2.1 (95 % CI 1.4–3.2)]”. The authors concluded that “one out of three women who suffer from psychological distress in late pregnancy with perceived social isolation will develop postpartum depression” (Forman et al. 2000).

Capioppo and Hawkley (2003) note that “social isolation is a potent but little understood risk factor for morbidity and mortality” with negative consequences “most profound among the elderly, the poor and minorities”. Social isolation may be seen as the antithesis of social support and connectedness. Indeed in introducing the phenomenon of “social isolation”, Capioppo and Hawkley draw on earlier meta-analysis of social support and connectedness (Uchino et al. 1996).

#### Abductive and retroductive analysis

Recontextualisation of perinatal depression within a Social Isolation conceptual framework provides explanation to the moderating effect of social support networks. Social isolation is the counterfactual of social support. Thus the extensive empirical research findings that associate “lack of social support” with perinatal depression give confidence to explaining the cause of perinatal depression in term of social isolation. Social isolation would not, however, be a necessary mechanism but would be a conditional cause of isolation and loneliness.

The structures that might give rise to social isolation as a causative mechanism might include: structure of work life, family structure, structure of the ethnic group, neighbourhood social structure, and religious group structure. Together these structures and mechanisms may explain the social isolation and loneliness of mothers with perinatal depressive symptoms.

As noted previously recontextualisation of the paradoxical multilevel findings within the social isolation conceptual framework suggest strong bonding networks in suburbs with low ethnic diversity contribute to social isolation of ethnic minorities.

Based on the triangulation, literature review and retroductive thought analysis we propose that the following conditions may influence the tendency of social isolation to cause isolation and loneliness.

1. No phone or visit support from midwife, nurse or other support worker
2. Poor partner or family support
3. Being part of an ethnic minority
4. Not having access to neighbours

**Fig. 4:**
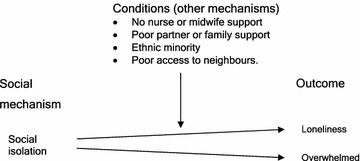
Critical Realist Model of Social Isolation Proposition

A critical realist model of the social level mechanism of social isolation is given in Fig. 4.

##### **Proposition**

*Social Isolation is a social level mechanism that causes maternal loneliness when certain contextual conditions exist.*

Assessment of Inference to the Best Explanation for the above propositions, using Hills “aspects of association” and Thagard’s principles and criteria, is shown (Table 3). In summary the social isolation proposition is not the best explanation. It explains some of the observed and known evidence but is insufficient on its own.

**Table 3:** Isolation propositions—inference to best explanation

Criteria	Application
*Hill’s aspects of association*	
Strength	Loneliness has strong QUAL assoc. & lack of support strong QUANT assoc. with depression
Consistency	The role of loneliness and lack of support has been found as a cause of depression has been found in large number of studies.
Specificity	No specificity identified
Temporality	No temporality demonstrated in this study
Biological gradient	Higher the lack of support the higher the observed depression
Plausibility	The association between loneliness and depression is biologically plausible
Coherence	The association is coherent with what is know
Experimental evidence	Interventions that provide support have been demonstrated to reduce depression
Analogy	There is an analogy between the effect of loss of expectation and loss of support. Both result in a similar effect
*Thagard’s Principles*	
Symmetry	There is symmetry between lack of support causing depression and support preventing depression
Explanation	The isolation proposition a) coheres with evidence on depression, b) evidence on role of support, isolation, loss of control, and c) is a single proposition
Analogy	Isolation causing stress is coherent with isolation causing depression mediated through stress
Data priority	Proposition describes the observation re isolation, support and depression
Contradiction	There are no contradictory proposals
Competition	No competitive explanation identified where p and q were not explanatorily connected
Acceptance	The isolation proposition is coherent with the overall system of propositions
*Thagard’s Criteria*	
Consilience	The central role of isolation as an explanation for stress and depression explains a range of facts but all known facts
Simplicity	Isolation is not sufficient to cause of depression. Not the most simple explanation
Analogy	Isolation causing stress in mothers is analogous to loss of control or expectations causing stress

### Social exclusion theory

#### Introduction

Marginalisation and poverty were identified as phenomenon associated with postnatal depression (Eastwood et al. 2015). The impact of social exclusion on psychological processes is reviewed by Hutchinson et al. (2007) who note that social exclusion causes “lowered self-esteem, greater anger, inability to reason well, depression and anxiety, and self-defeating perceptions and behaviours”. Bonner (2006) notes that the most common mental health problem experienced by socially excluded people is depression. We will redescribe and recontexualise the phenomena of marginalisation and poverty associated with postnatal depression within the social level concept of social exclusion. The central purpose in this section is to explain the phenomenon of postnatal depression within the social exclusion conceptual framework.

#### Triangulation

The possibility of social exclusion as a generative mechanism of the phenomenon of postnatal depression emerged as the concept of “marginalisation” in the individual level qualitative study (Eastwood et al. 2015). The literature review (Eastwood 2011) and individual level quantitative study (Eastwood et al. 2012a, b) both provided empirical evidence that financial difficulties, accommodation, unemployment, sole parenthood and access to a car were risk factors for postnatal depression.

The individual level qualitative study (Eastwood et al. 2015) identified “being broke” as a mechanism that could result in stress and depression. Also identified were unplanned pregnancy, sole parenthood, maternal education, access to phone and cars, “poverty”, class, social position and social hierarchy. The group level qualitative study (Eastwood et al. 2014b) identified living in a depressed community was a strong theme. The situational analysis identified the global economy as playing an important role in relation to economic marginalisation and social exclusion. As noted above there was a link of social exclusion with social isolation, social networks, social cohesion and social capital (Eastwood 2011).

The group level quantitative study (Eastwood et al. 2014) included a number of indicators of social exclusion. The Exploratory Factor Analysis loaded the following variables on Factor 1: sole parenthood, home rental, public accommodation, unplanned pregnancy, low occupational class, poor, raised crime rate, Index or Relative Social Deprivation (IRSD) Deciles, not being in a single dwelling and self-reported poor health (Eastwood et al. 2014d). Visualisation and bivariate analysis found an association between most measures of social exclusion and the aggregated and spatial distribution of postnatal depression (Eastwood 2011). Bayesian bivariate analysis was consistent. In the ecological linear regressions “Occupation Class 3 %” and “Home Ownership %” were significant for EDS > 12 (Eastwood et al. 2013). The Bayesian spatial analysis found no measures of social exclusion were significant (Eastwood et al. 2014).

The Bayesian multi-level spatial analysis found for EDS > 12 the Index of Concentrated Extremes (ICE), Index of Relative Social Disadvantage (IRSD), % Poor and Factor 1 improved the model but were not significant (Eastwood 2011). In the stratified multilevel analysis measures of social exclusion did not improve the model for mothers born in Australia but for those mothers not born in Australia ICE, IRSD, % Occupational Class 3 and % unemployed improved the model for EDS > 9. Paradoxically a high percentage of occupation class 3 and unemployment protected against depression. None of the measures of social exclusion were significant in the non-born in Australia EDS > 12 analysis (Eastwood et al. 2013c).

The ecological studies (linear regression and spatial) did not find a strong association of aggregated postnatal depression with measures of social exclusion. The exception was apartment living. Similarly when controlling for individual level factors (including financial difficulties) the multilevel studies only found a significant group level effect in the “not born in Australia” EDS > 9 study (Eastwood et al. 2013c).

The quantitative findings suggest that social exclusion is operating predominantly at the individual family level with the exception of mothers not born in Australia where it is impacting on less severe depressive symptomatology (EDS > 9). For mothers not born in Australia living in communities with extremes of wealth is detrimental to mental health, while living in communities with a high percentage of labourers, clerical workers and unemployed is protective (Eastwood 2011).

An alternative explanation is that social exclusion is operating distal to social support in a causal chain. Consequently both the spatial and multilevel regressions will control for social exclusion and the only significant associations are with the more proximal social support measures.

Recontextualisation of these multilevel findings within the social exclusion conceptual framework suggests that for migrant mothers, social relations (and possible bridging networks) are stronger in working class communities and protect against maternal depression. The converse logic is that, for migrant mothers, there is more social exclusion than might be expected, in suburbs with extremes between rich and poor. Interestingly these findings were not apparent for mothers born in Australia.

As previously discussed, women of low socio-economic status (SES) have consistently been found to have higher rates of antenatal and postnatal depression (O’Hara 1995; O’Hara et al. 1984; Cox et al. 1982; Gotlib et al. 1991; Zajicek 1981; Bennett et al. 2004; Seguin et al. 1999; O’Hara and Swain 1996; Beck 2001). Beck, in her first meta-synthesis (Beck 1996), did not find SES to be significant but added both SES and unplanned pregnancy in her later update (Beck 2001). The relationship between SES and postpartum depression, as reported in the 2001 meta-analysis, was a small effect size (0.19 ~ 0.22).

There is increasing evidence for the area-level contextual effects of economic deprivation on mental health (Fone and Dunstan 2006; Fone et al. 2007a, b; Weich et al. 2002, 2003; Skapinakis et al. 2005). O’Campo et al. (2009) in their concept mapping of pathways from neighbourhoods to mental well-being found that “neighbourhood affordability, negative community factors including crime and vandalism, and social makeup including unemployment and poverty, were felt to be associated with poor mental well-being” (O’Campo et al. 2009).

The definition of social exclusion remains contested but there is a common “understanding that social exclusion is not only about material poverty and lack of material resources, but also about the processes by which some individuals and groups become marginalised in society” (Millar 2007). While there is an overlap with the concept of social isolation, it is used here primarily in relation to access to resources.

In a framework of social exclusion indicators Tsakloglou and Papadopoulos (2002) grouped the indicators into “measures of: income poverty; living conditions; necessities of life; and social relations. The measures of social relations included meeting friends, talking to neighbours and membership of clubs or groups”. It is worth noting here that the inclusion of measures of social relations will result in an overlap, or correlation, with measures of social isolation, social cohesion, social support and social capital that are discussed separately. Saunders (2003) undertook a study using the 1998/99 Household Expenditure Survey (HES) to assess the level of social exclusion in Australia. Saunders studied the following major forms of social exclusion: social interaction, domestic deprivation and extreme consumption hardship. He found that the major form of social exclusion was lack of social interaction which he measured at twice the level of domestic deprivation and four times the level of extreme consumption hardship. Saunders also found that sole parents were the most excluded group. In a comparative study between Australia and Britain, Sanders and Adelman (2005) found that poverty was higher in Britain but levels of material deprivation and social exclusion were higher in Australia, with sole parents the most affected in both countries. The finding by Saunders that lack of social interaction was the major form of social exclusion in the Australian setting is consistent with the emerging findings from this study. Social exclusion may be impacting on mothers through social isolation and social support rather than material deprivation.

#### Abductive and retroductive analysis

Redescribing poverty and marginalisation within a social exclusion conceptual framework provides explanation for a possible causative mechanism called social exclusion. Social exclusion is a contested notion about the process by which some individuals and groups become marginalised from society (Millar 2007). We have abstracted that social exclusion may impact on mothers through social isolation and stress. While there may be many causes of social isolation social exclusion is clearly an important social level mechanism.

Structures within the social level that may generate social exclusion as a causative mechanism include: global markets, big business, political structures, dominant culture, government agencies, religious organisations, and capitalist structures. Candidate conditional mechanisms may include: income support policy, ethnic segregation or diversity, racial tolerance, religious tolerance, public transport, and social cohesion.

As noted above recontextualisation of paradoxical multilevel findings within the social exclusion conceptual framework suggests that for migrant mothers, social relations (and possible bridging networks) are stronger in working class communities and protect against maternal depression. The converse logic was that, for migrant mothers, there is more social exclusion than might be expected, in suburbs with extremes between rich and poor. As previously noted, these findings were not apparent for mothers born in Australia.

Based on the triangulation, literature review and retroductive thought analysis we propose that the following conditional mechanisms may influence the tendency of social exclusion to cause social isolation, stress and depression:

1. weak bridging networks or social cohesion
2. segregation of social and ethnic groups and strong bonding networks
3. poor access to services including home visiting nursing services.

**Fig. 5:**
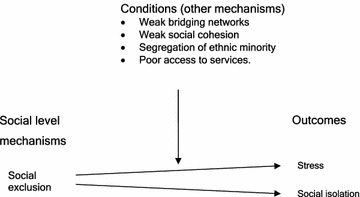
Critical Realist Model of Social Exclusion Proposition

A critical realist model of the social level mechanism of social exclusion (Fig. 5).

##### **Proposition**

*Social exclusion is a social level mechanism that causes maternal stress and isolation when certain personal characteristics and contextual conditions exist*

Assessment of Inference to the Best Explanation for the above propositions, using Hills “aspects of association” and Thagard’s principles and criteria, is shown (Table 4). In summary the social exclusion proposition is not the best explanation. It explains some of the observed and known evidence but is insufficient on its own.

**Table 4:** Social exclusion proposition—inference to best explanation

Criteria	Application
*Hill’s aspects of association*	
Strength	Financial stress has a strong assoc with depression at the individual level but ecological measures of poverty and social exclusion had weak association
Consistency	The poor income has been found as a cause of depression in large number of studies
Specificity	No specificity identified
Temporality	No temporality demonstrated in this study
Biological gradient	Higher the lack of financial stress the higher the observed depression
Plausibility	The association between social exclusion and stress is biologically plausible
Coherence	The association is coherent with what is know
Experimental evidence	No experimental evidence identified
Analogy	There is an analogy between the effect of social exclusion and isolation. Both result in a similar effect
*Thagard’s Principles*	
Symmetry	There is symmetry between social exclusion causing depression and social support and practical support preventing depression
Explanation	The social exclusion proposition a) coheres with evidence on depression, b) coheres with other propositions and c) is a single proposition
Analogy	Social exclusion causing stress is coherent with isolation causing depression mediated through stress
Data priority	The proposition describes the observation re financial stress and depression
Contradiction	There are no contradictory proposals
Competition	No competitive explanation identified where p and q were not explanatorily connected
Acceptance	The social exclusion proposition is coherent with the overall system of propositions
*Thagard’s Criteria*	
Consilience	Social exclusion explains a range of facts but not all known facts
Simplicity	Social exclusion is not sufficient to cause of depression. Not the most simple explanation
Analogy	Social exclusion causing stress in mothers is analogous to social exclusion causing poor self report health

### Social service mechanisms

#### Introduction

Access to services was identified by mothers as a phenomenon associated with protecting mothers from postnatal depression (Eastwood et al. 2015). The buffering impact of delivery of social services on maternal (and infant) psycho-social and psychological processes is reviewed by Hutchinson et al. (2007). Here we use abductive logic to redescribe and recontexualise the phenomena of access to services associated with postnatal depression within the policy level concept of delivery of social services. The central purpose in this section is to ascribe meaning to the phenomenon of postnatal depression within a social services conceptual framework.

#### Triangulation

The possibility of that social services may play a protective role in the phenomenon of postnatal depression emerged in the individual level qualitative study (Eastwood et al. 2015) where mothers spoke of the supportive role of midwives, nurses, mother’s groups and phone calls. The literature review (Eastwood 2011) did not identify lack of social services as a risk factor for postnatal depression. There is evidence that service interventions can play a role in treating postnatal depression (Morrell et al. 2009; Dennis et al. 2009; Dennis 2010). The individual level quantitative studies did not include variables that measured levels of social service delivery. Any additional nurse visits would have occurred after the EDS was measured.

Group level theoretical and empirical literature suggests that the social service environment may play an important role in influencing perinatal outcomes (Culhane and Elo 2005). The group level qualitative study identified “access to services” and “supportive social policy” as important themes (Eastwood et al. 2014). The situational analysis drew these together into a combined services planning, delivery and social policy arena (Eastwood 2011, pp. 203–210).

The group level quantitative study (Chapter 8) included two measures of early childhood nursing support. Analytical findings were mixed. The Exploratory Factor Analysis loaded both the variables on Factor 5 together with % poor health and % different address in 5 years (Eastwood et al. 2014). Spatial visualisation suggested an association between nursing visiting rates and aggregated EDS but the bivariate analysis, while statistically significant, had low R Squared. Bayesian bivariate analysis was not significant. In the ecological linear regressions “Nurse Visit Rate %” was significant for both EDS > 9 and EDS > 12 but did not contribute to improving the Bayesian ecological spatial models (Eastwood et al. 2013). In the Bayesian multi-level spatial studies nursing visiting rate improved, but not significantly, the EDS > 9 overall and not-born in Australia studies. Factor 5 also improved the same models but was not significant (Eastwood 2011; Eastwood et al. 2013).

The quantitative studies had a limited set of variables for measuring service access. Visualisation showed marked spatial differences in nursing service delivery which correlated with the distribution of aggregated EDS > 9 and EDS > 12. The analytical studies also suggested a possible contribution to both the aggregated and individual level EDS rates.

Recontextualisation of these multilevel findings within the social services policy conceptual framework suggests that services policy and resourcing may contribute to protecting against maternal depression through social and psycho-social level mechanisms.

There is limited evidence, however, that service level interventions will prevent the risk of postnatal depression. We have previously reported (Eastwood et al. 2012b) that Dennis (2005) undertook a systematic review of all published and unpublished randomised controlled trials of preventive psychosocial and psychological interventions in which the primary or secondary aim was a reduction in the risk of postnatal depression. All the trials recruited pregnant women or new mothers less than 6 weeks postpartum. Of the fifteen trials assessed the only intervention to have a clear preventative effect was intensive postpartum support provided by a health professional. It was notable that individually based interventions were more effective than group based interventions. The efficacy of home visiting for postnatal depression has recently been confirmed (Morrell et al. 2009). Social support networks were protective in this study suggesting that antenatal interventions that promote friendship groups may be beneficial. The role of antenatal groups in preventing postnatal depression has not yet been confirmed (Austin 2003).

More recent studies have, however, demonstrated the effectiveness of support-focused interventions by social services. Dennis et al. (2009) undertook a multisite randomised controlled trial where the intervention was proactive individualised telephone based peer (mother to mother) support, provided by a volunteer recruited from the community who had previously experienced and recovered from self-reported postnatal depression and who had attended a 4 h training session. Relationship-based community health worker support through phone and face to face contact was demonstrated, in a randomised control trial, to alleviate depressive symptoms in Medicaid eligible women compared to controls. The effect was greater for women at higher risk (Roman et al. 2009).

Broader social service interventions that are often described as “joined-up Government” may also have benefits. “Joined up” interagency approaches to improving child and family health, such as UK Sure Start initiative have had mixed results (Rutter 2006). The recent study by Mulhuish et al. (2008) however, found promising outcomes not identified in earlier evaluations. While these outcomes were among children, the interventions were family focused and many commenced during pregnancy and are thus relevant to this analysis.

#### Abductive and retroductive analysis

The redescription of maternal depression within a services conceptual framework focuses analysis on the service structures that may generate causative mechanisms. That analysis identifies a complex set of structures and mechanisms that is beyond the scope of this study. For example, service delivery arises from political, public service, health service, business and economic structures.

Simplistically social service mechanisms may provide interventions that reduce social exclusion, social isolation, stress and depression. Those mechanisms arise at macro and meso levels but may impact directly on biological and psychological levels. For illustration we have included the following conditional mechanisms in the model.

1. Political support for social service interventions
2. Public Service Policy level support for social service interventions
3. Operation level support for effective evidence-based social service interventions.

**Fig. 6:**
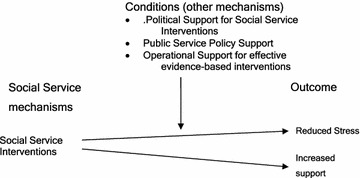
Critical Realist Model of Social Service Proposition

A critical realist model of the social level mechanism of social services is given in Fig. 6.

##### **Proposition**

*Social Services Delivery is a policy level mechanism that protects against maternal stress and isolation when certain other personal characteristics and contextual conditions exist.*

Assessment of Inference to the Best Explanation for the above propositions, using Hills “aspects of association” and Thagard’s principles and criteria, is shown (Table 5). In summary the social service proposition is not the best explanation. It explains some of the observed and known evidence but is insufficient on its own.

**Table 5:** Social service proposition—inference to best explanation

Criteria	Application
*Hill’s aspects of association*	
Strength	Social services were identified by QUAL but has a weak association with depression at the ecological level. No individual level data available
Consistency	The Social services have not been well studied as protective also intervention studies show some effect
Specificity	No specificity identified
Temporality	No temporality demonstrated in this study
Biological gradient	Limited information available
Plausibility	The association between Social Services and stress is plausible
Coherence	The association is coherent with what is know
Experimental evidence	There is experimental evidence that service intervention can reduce depression
Analogy	There is an analogy between of social service support with social support and practical providing buffering
*Thagard’s Principles*	
Symmetry	There is symmetry between social services buffering stress and social support and practical support preventing depression
Explanation	The social service proposition a) coheres with evidence on depression, b) coheres with other propositions and c) is not a single proposition
Analogy	Social services buffering stress is coherent with social support buffering stress
Data priority	The proposition describes the observation re ecological association
Contradiction	There are no contradictory proposals
Competition	No competitive explanation identified where p and q were not explanatorily connected
Acceptance	The social service proposition is coherent with the overall system of propositions
*Thagard’s Criteria*	
Consilience	Social service proposition explains a limited range of known facts
Simplicity	Social service proposition is not sufficient to protect from depression. Not the most simple explanation
Analogy	Social service buffering stress in mothers is analogous to social support buffering stress and depression

### Social capital theory

#### Introduction

Maternal emotional, practical and social support networks were identified as phenomenon protecting mothers from postnatal depression (Eastwood et al. 2012a, b, 2015). We will use abductive logic here to redescribe and recontexualise the phenomena of support associated with postnatal depression within the psycho-social and social level concepts of social networks and social capital. The central purpose in this section is to ascribe meaning to the phenomenon of postnatal depression within a social capital conceptual framework.

Kawachi et al. (2008) argue that social capital has both individual and group attributes and that it can be conceptualised as both social cohesion and as resources embedded in networks. The idea that social capital has both individual and group attributes is consistent with the findings in this study which has found network-based resources to be important at both the individual and group levels. The authors also observe that there is currently contention as to whether social capital should be conceptualised as social cohesion or as resources imbedded in social networks.

Moore et al. (2006) advanced the argument that network approaches to social capital were lost when the concept was translated into public health. The authors argue that social capital was originally viewed within public health as a psychosocial mechanism operating at an ecological level that might mediate the income inequality-health pathway. This led to a dominance of what the authors call a “communitarian” approach to social capital with “disproportionate attention to normative and associational properties of places”. They argued that network approaches to social capital should be recovered in order to enable the full translation and conceptualisation of social capital in public health [and epidemiology] (Moore et al. 2006).

Hulse and Stone (2007), writing from an Australian policy perspective, reviewed the use of the terms social cohesion, social capital and social exclusion across North American, European and Australasian jurisdictions. In their account they identify at least “three dimensions of social cohesion:

1. Social relations of everyday life, family and social relationships, networks, and voluntary social processes (social capital)
2. Reduction of differences, cleavages and inequalities between groups of people and people living in different geographical areas (social exclusion)
3. A distinct cultural dimension referring to “ties that bond people together with a sense of common purpose, shared identity and common values (social cohesion)”.

This debate is well articulated by Carpiano (2006, 2008) who compares two theories of social capital as advanced by Putman (1993, 1995, 2000) and Bourdieu (Bourdieu 1986; Bourdieu and Wacquant 1992). Carpiano (2008) argues for the separation of what he calls “the: (1) structural antecedent factors (i.e., socio-economic conditions, residential stability, income inequality), (2) social cohesion (connectedness and values), (3) social capital (social support, social leverage, information social control, neighbourhood organisation participation), and (4) social capital outcomes” (Carpiano 2008).

Of relevance to the analysis here are the bonding and bridging forms of social capital. Kawachi et al. (2008) observe that “regardless of whether one subscribes to the social cohesion school of social capital or the network school, consensus now exists about the importance of distinguishing between bonding and bridging social capital”. Bonding social capital refers to resources that can be accessed within social groups whose members are alike in terms of their social identify. The term “bridging capital” is used to describe the process whereby resources are accessed by individuals and groups through their connections that cross class, race, cultural and other boundaries of social identity. Bonding capital may have detrimental effects and a key to improving health may be increasing access to resources outside of immediate social milieu (Kawachi et al. 2008).

#### Triangulation

The possibility of social capital as a generative mechanism of the phenomenon of postnatal depression emerged as the concept of “support and nurturing” of mothers in the individual level qualitative study (Eastwood et al. 2015) and the subsequent group level qualitative study (Eastwood et al. 2014b). The individual level literature review and quantitative study (Eastwood et al. 2012a, b) provided empirical evidence that social relationships are protective for maternal postnatal depression. The group level findings were paradoxical and suggest that ethnic diversity is playing a significant role (Eastwood et al. 2013c).

The individual level qualitative study (Eastwood et al. 2015) identified “support for mothers” as a mechanism that could protect mothers from stress and depression. Also identified were partner support, family support, support of a “mum type” person, social networks, and mothers groups. The group level qualitative study (Eastwood et al. 2014b) identified social capital as a strong theme and the situational analysis confirmed the importance of the linked concepts of social support networks, social cohesion and social capital in protecting mothers from depression.

The individual level logistic regression (Eastwood et al. 2012a) identified measures of emotional support, practical support and social support as being significantly associated with EDS > 9 and EDS > 12. The group level quantitative studies included a number of indicators of social capital. In relation to Social Capital, the Exploratory Factor Analysis (Eastwood et al. 2014d) loaded the following variables on Factor 2: Volunteerism, Low lack of schooling, high entropy, low percentage of Class 3, High IRSD, low density, high nurse visiting rate; and Factor 6: low lack of schooling, low % no social support, low % no practical support, low density, high different address in past five years and high Maly index (2000). Both these latent variables included measures of ethnic diversity.

Ecological visualisation and bivariate analysis found associations between most measures of social networks, social cohesion and social capital and the aggregated and spatial distribution of postnatal depression. Bayesian bivariate analysis was consistent except for measures of change of address. In both the ecological likelihood linear and Bayesian spatial regressions “% No Support” and “Entropy” were significant for EDS > 9 and EDS > 12 (Eastwood et al. 2013a).

The Bayesian multi-level spatial analysis found for EDS > 12 the density, the Maly Index and Factor 2 improved the model but were not significant. Low % no support, low % poor schooling and Factor 6 improved the model and were significant. Factor 6 significantly improved the Bayesian multi-level model reducing the DIC from 5215 to 5209 (Eastwood 2011, p. 289). In the stratified multilevel analysis measures of social capital did not improve the model for mothers born in Australia but for those mothers not born in Australia ICE, Maly Index, % no support and % poor schooling improved the model for EDS > 9 and EDS > 12 (Eastwood et al. 2013c). These findings are paradoxical and suggesting that low group level social capital may be protective when controlling for individual level measures of support.

The ecological studies (linear regression and spatial) found a strong association of aggregated postnatal depression with aggregated measures of social support. When controlling for individual level factors (including social support) the multilevel studies found a significant protective group level effect of “no social support”. In the stratified studies this finding was not found for mothers born in Australia. For mothers not born in Australia this finding was associated with a group level protective effect from increased neighbourhood ethnic diversity (Maly Index) (Maly 2000).

The multi-level quantitative findings suggest that social capital is operating predominantly at the individual family level with the exception of mothers not born in Australia. For mothers not born in Australia living in communities with high levels of social capital (social networks) is detrimental to mental health.

Recontextualisation of these multilevel findings within the social capital conceptual framework suggests that for migrant mothers, social relations (and possible bridging networks) are stronger in ethnically diverse communities and protect against maternal depression. The converse logic is that, for migrant mothers, there is more social support than might be expected, in suburbs with low aggregated social support. Interestingly these findings were not apparent for mothers born in Australia.

There limited literature in relation to perinatal depression and social capital. There is, however, extensive empirical evidence for the role of the social support component of social capital as described by Carpiano (2008). Social support has also been found to be protective of a wide range of other adverse perinatal outcomes (Orr 2004).

Social support is a concept with multiple dimensions. Support can be from a spouse, relatives, friends, or associates. Social support can be defined as being of three types: (1) *informational support* (where advice and guidance is given); (2) *instrumental support* (practical help in terms of material aid or assistance with tasks); and (3) *emotional support* (expressions of caring and esteem).

Emotional support can be defined as “relationships that make the individual feel love, appreciated, and valued. Instrumental support has been described as tangible assistance and includes support available to the individual from members of his or her family or others to help with specific, concrete needs, such as lending money, giving a ride, helping with childcare etc.” (Orr 2004).

Social support and its counterfactual, lack of social support, have consistently been found to be associated with perinatal depressive symptoms (O’Hara 1995; O’Hara et al. 1984; Cox et al. 1982; Gotlib et al. 1991; Zajicek 1981; Bennett et al. 2004; Seguin et al. 1999; O’Hara and Swain 1996; Beck 2001). Beck (2001) identified 27 studies that examined social support and postpartum depression. The relationship between social support and postpartum depression had a moderate effect size. Other studies have consistently shown a negative correlation between postpartum depression and emotional and instrumental support during pregnancy (Beck 1996; O’Hara and Swain 1996; Seguin and Potvin 1999; Menaghann 1990; Richman et al. 1991; Ritter et al. 2000).

Social support might be particularly relevant in relation to stressors experienced by immigrants. Surkan et al. (2006) found that social support, and social networks were independently related to depressive symptoms in a multiethnic sample of women having recently given birth.

Ritter et al. (2000) cites House et al. (1988) as noting that “social support may have a more positive direct effect on health than stress has a negative effect”. They also note that social support has been found to limit the negative psychological effects of chronic life stress. In the relation to pregnancy, social support has been found to have a positive effect on psychological well-being even among low income women. Ritter et al. (2000) argue that “because of the relative lack of financial resources, low-income pregnant women may be particularly reliant on social support, because they may become less likely to work and have greater needs for emotional and emotional assistance”. They note, however, that this positive effect may not apply to inner-city or ethnic minority women where “women’s social resources may already be stretched owing to chronic stressful conditions and may no longer be available during stressful periods” (Ritter et al. 2000).

The impact of social capital on mental health has been recently reviewed by Almedom and Glandon (2005, 2008). Those reviews found that social capital could be both and “asset and a liability with respect to mental health of those in receipt of and those providing services and other interventions”. The review included both individual level and ecological level studies and none of the studies reviewed were in relation to perinatal mental health.

De Silva et al. (2005) in a systematic review of 21 studies suggested that individual and ecological studies of social capital may measure different aspects of the social environment. They concluded that current evidence is inadequate to inform the development of specific social capital intervention to combat mental illness. Their review included 7 ecological (6 multilevel) studies none of which related specifically to perinatal maternal mental health. With respect to the ecological studies there was no clear pattern of association between ecological social capital and mental illness. In particular there was no clear evidence of an inverse relationship between the level of social capital and mental illness.

Recent multi-level studies lend some support to the hypothesis that ecological level social capital may protect against mental health disorders. A UK multilevel study by Fone (2007) found that “small-area level income deprivation and low levels of social cohesion were independently associated with poor mental health” and that “high levels of social cohesion attenuated the association between mental health and income deprivation”. In another UK multilevel study Stafford et al. (2008) found no evidence of a main effect of social capital on mental health. For people living in deprived circumstances, however, an association between social capital (contact amongst local friends) and lower reporting of common mental disorders was found. Of relevance to the findings of this study was the finding that elements of bonding social capital were associated with higher reporting of common mental disorders. This finding was only in deprived households.

Bonding capital may represent “an important survival mechanism for residents of disadvantaged communities” (Kawachi et al. 2008). Kawachi and colleagues cite Stack’s (1974) ethnographic study of a poor African-American community which revealed high levels of mutual support from kinship networks as the primary mechanism for “getting by”. The authors observe that such “bonding capital often extracts a cost to the providers of support in terms of the mental and financial strain of caring for others in need”. Kawachi and colleagues cite other studies that also (Caughy et al. 2003; Ziersch and Baum 2004) suggest that strong bonding ties within disadvantaged communities may be a detriment to the health of residents. The authors conclude that “the emerging picture from these studies seems to be that bonding capital within disadvantaged communities may be a health liability rather than a force for health promotion that it is often assumed to be. The key to improving health therefore appears to lie in residents’ ability to access resources outside their immediate social milieu, i.e., access to bridging social capital” (Kawachi et al. 2008).

#### Abductive and retroductive analysis

The redescription of maternal depression within a social capital conceptual framework focuses analysis on social network structures that may generate causative mechanisms. Those mechanisms may be protective or detrimental depending upon the context. In this study emotional and practical support and social networks all had protective effects at the individual level. Put simplistically these modes of support may reduce the effects of social exclusion, social isolation, stress and depression. The social capital mechanisms may arise at macro and meso levels but are impacting directly on the individual psychological level.

The outcome of this social capital is usually a reduction in stress but in some circumstances there may be an increase in stress. The forms of social capital are complex and cannot be reduced to a single construct. We have therefore considered that bonding, bridging and linking social ties should be considered as separate social capital mechanisms.

The contextual conditions are poorly understood but based on the findings in this study and the above literature we have included the following conditional mechanisms in the model:

1. Class and social position
2. Poverty and social exclusion
3. Crime and community safety
4. Neighbourhood decay
5. Population density
6. Social service support

**Fig. 7:**
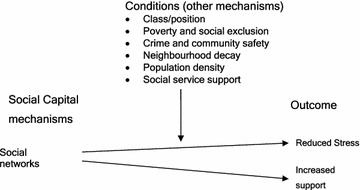
Critical Realist Model of Social Capital Proposition

A critical realist model of the social capital mechanism is below (Fig. 7).

Based on the above analysis the following proposition is made:

##### **Proposition**

*Social capital (social networks) is a social level mechanism that protects against maternal stress and isolation when certain other personal characteristics and contextual conditions exist*

Assessment of Inference to the Best Explanation for the above propositions, using Hills “aspects of association” and Thagard’s principles and criteria, is shown (Table 6). In summary the social service proposition is not the best explanation. It explains some of the observed and known evidence but is insufficient on its own.

**Table 6:** Social capital proposition—inference to best explanation

Criteria	Application
*Hill’s aspects of association*	
Strength	Social Capital (social networks) was identified by QUAL and QUANT as associated with depression at the individual and ecological levels
Consistency	Social Capital (social networks) and social support has been previously identified as associated with maternal depression
Specificity	Postnatal depression is strongly linked to lack of support
Temporality	No temporality demonstrated in this study
Biological gradient	There was a gradient at the individual and ecological level
Plausibility	The association between social networks with stress is plausible
Coherence	The association is coherent with what is know
Experimental evidence	There is experimental evidence that home visiting and other support reduced maternal depression
Analogy	There is an analogy between of social network support and the benefits of emotional and practical support all reducing stress and depression
*Thagard’s Principles*	
Symmetry	There is symmetry between social capital reducing stress and social isolation causing stress
Explanation	The social capital propositions a) coheres with evidence on depression, b) coheres with other propositions and c) is not a single proposition
Analogy	There is an analogy between of social network support and the benefits of emotional and practical support all reducing stress and depression
Data priority	The proposition describes the data observations
Contradiction	There are no contradictory proposals
Competition	No competitive explanation identified where p and q were not explanatorily connected
Acceptance	The social capital propositions are coherent with the overall system of propositions
*Thagard’s Criteria*	
Consilience	Social capital propositions explain a limited range of known facts
Simplicity	Social capital propositions are not sufficient to explain depression. Not the most simple explanation
Analogy	There is an analogy between of social network support and the benefits of emotional and practical support all reducing stress and depression

### Global-economic level mechanisms

#### Introduction

The intention was to limit this study to neighbourhood and community level economic, social and physical influences. Clark challenges this perspective and argues “everything in the situation both constitutes and affects most everything else in the situation in some way … Here the macro/meso/micro distinctions dissolve in the presence/absence” (Clarke 2005).

The critical realist ontology provides another perspective by claiming that reality is stratified and that each level can influence each other level. The levels are defined by the structures and their generative mechanisms. Table 1 described the following ontological levels as relevant to this study: biological, psychological, social, cultural and global economic. We have limited the analysis in this manuscript to the social level with some exploration of psychological and cultural Levels. Here we will provide brief analysis of a number of global-economic level mechanisms identified from the qualitative studies and associated retroductive analysis (Eastwood et al. 2014b).

1. Migration
2. Media
3. Big Business and Power.

#### Migration

Migration has been a significant historical feature of the populating of South West Sydney. Following the Second World War those migrants originated predominantly from Europe. Since the 1970s there has been a significant migration of Indo-Chinese peoples and more recently migration of peoples from Eastern Europe and the Middle East. In this study approximately forty five percent of mothers were not born in Australia. The role of migration and acculturation will be included as a Global-Economic Level mechanism in the development of the theoretical conceptual framework. Analysis is at Additional file 1: Appendix 1.

#### Media and advertising

The role that media and advertising might play in relation to perinatal depression and maternal expectation emerged from focus groups and situational analysis in the individual level qualitative study (Eastwood et al. 2015). “Huggies adverts” were used to describe what Beck called the “incongruity between expectations and the reality of motherhood”. Another impact that media and advertising may have on mothers relates to lifestyle “dreams”. These dreams and aspirations relate to aspirations that she may have regarding wealth, material goods, holidays and education for her children. The large shopping malls with their “glitter” were seen as also playing a role. This portrayal of mothers, babies, “motherhood” and lifestyle “dreams” in advertising may reflect existing cultural norms, or alternatively play a role in creating expectations that may be inaccurate. We have postulated that media and advertising constitute a generative mechanism that is influencing mother’s expectations and thus contributing to stress.

“Big Media” in Australia plays an important role in relation to sport franchises. Emerging from the interviews was concern regarding the impact that this had had on the local community when the local Rugby League team merged with a team from outside the region. Rugby League in South West Sydney was reported to have declined with no obvious replacement sporting outlet for young people. The Malls with their “glitter” and the loss of the local football were both seen as contributing to the phenomenon of “depressed community”. We have postulated that media and advertising thus constitute a generative mechanism that contributing to a “depressed” neighbourhood context.

Media and Advertising will be included as Global-Economic Level mechanisms in the development of the theoretical conceptual framework.

#### Big business and power

The strong influence of big business, media and the global economy on the situation for mothers and infants emerged from both qualitative studies (Eastwood et al. 2014b, 2015). We have previously observed that “the impact of the large shopping malls on local communities was seen as both having positive and negative impacts on mothers as it provided meeting places for mothers but also “depopulated” local shopping areas and parks” (Eastwood et al. 2014b).

Big business was seen as having “power” to influence local politicians. One example given, related to the building of “fast food” outlet on land that community members wished to use as a community garden. The “McDonaldization” thesis advanced by Ritzer (2008, p. 457) “is the process by which the principles of the fast-food restaurant are coming to dominate more and more sectors of society”. Ritzer describes the process as being delineated “by efficiency, calculability, predictability, control through technology and ‘irrationality of rationality’”. The later process inevitably leads to dehumanisation of jobs, settings and circumstances (Ritzer 2008, p. 459) with impacts on local neighbourhoods and communities.

During the course of this study, South West Sydney experienced the impact of global economic mechanisms with closure of large business and loss of employment. Corporate Business elected to move industry to other countries and jurisdictions. As a result the New South Wales Government budgets were affected leading to impacts on urban development, social services and maintenance of essential infrastructure. We have postulated that corporate business is a global-economic level structure with generative powers that can have significant impacts on the neighbourhood context. Corporate Business will be included as a Global-Economic Level mechanism in the conceptual framework that follows.

### Comparison of theories

#### Introduction

Making a comparison between different theories and abstractions is Stage 5 of the critical realist “stages in explanatory research” as described by Danermark et al. (2002, p. 110). “In this stage one elaborates and estimates the relative explanatory power of the mechanisms and structures which have been described by means of abduction and retroduction within the frame of stages 3 and 4”. As suggested by Danermark and colleagues we initially undertook this process as part of the abductive and retroduction abstraction as in the previous sections.

#### Contribution from the analysis

The theories used for that analysis are complementary and focus on different mechanisms and context. The retroductive analysis across sections remained consistent. Areas of complexity and uncertainty, such as the role of bonding and bridging social capital, were similar for each of the theoretical perspectives and were consistent with findings from empirical studies.

No one theoretical perspective was able to provide a complete explanation of neighbourhood context and the phenomenon of perinatal depression. Stress Process theory provided a strong foundation for building a conceptual framework of maternal depression, stress and neighbourhood context. Theories of social isolation, exclusion, and capital were able to strengthen the explanatory power of the emerging framework.

Missing from analysis at the psychological and social levels was the explanatory power provided by a study of culture. The analysis of acculturation and ethnic segregation contributed significant to explaining the study findings. But this analysis did not fully explore the role of mainstream Australian “culture”. The brief analysis of media and advertising argued that advertising was a mechanism that influenced maternal expectations of motherhood. We have elected to not to explore theory related to media in further depth here.

#### Contribution from other theories

We reviewed a number of related theories that might inform the construction of a theoretical framework of maternal depression and neighbourhood context. That review can be found at Additional file 2: Appendix 2.

The “eco” theories critically examined by Krieger (2001) have played an important role in the development of social epidemiology theory. This layered approach has been conceptually used for the development of recent multi-level studies based on spatial or areal units. This approach also forms the basis for the conditional matrix proposed by Corbin and Strauss (2008, p. 94).

The approach is, however, overly simplistic and as Clarke observes “everything in the situation both constitutes and affects most everything else in the situation in some way”. Such a view is consistent with critical realism ontology where each strata may interact with layers above and below to produce new mechanisms, objects and events. Critical realism also places importance on temporal and spatial dimensions. The eco-social construct proposed by Krieger (2001) is similar to critical realism and draws on the concept of embodiment which incorporates the biological, material and social worlds.

The psychosocial theory is central to the development of the conceptual framework developed here and is closely related to the stress process model proposed by Pearlin (1989). As Krieger observes, however, the psychosocial theory does not explain who and what generates the psychosocial insults and buffers or how they are distributed (Krieger 2001). This is where critical realist with its examination of structures and mechanisms can contribute.

The theoretical models of neighbourhood effects (Ellen et al. 2001; Macintyre and Ellaway 2003) are directly relevant to this study. The emerging theory is consistent with that proposed by Ellen et al. (2001) with the identification of social stresses and the buffering effects of social networks. Neighbourhood or community institutions, resources and physical attributes emerged from the qualitative studies as important stressors or generative mechanisms.

The perinatal models (Matthews and Meaney 2005; Culhane and Elo 2005; Misra et al. 2003) reviewed above are consistent with the emerging conceptual framework. In particular, the model described by Matthews and Meaney (2005) goes a long way to describing the findings of this study.

Finally Becks’ metasynthesis and model provides a explanation of the mechanisms and processes occurring at the psychological level and will be included in the conceptual framework that follows (Beck 2002).

**Fig. 8:**
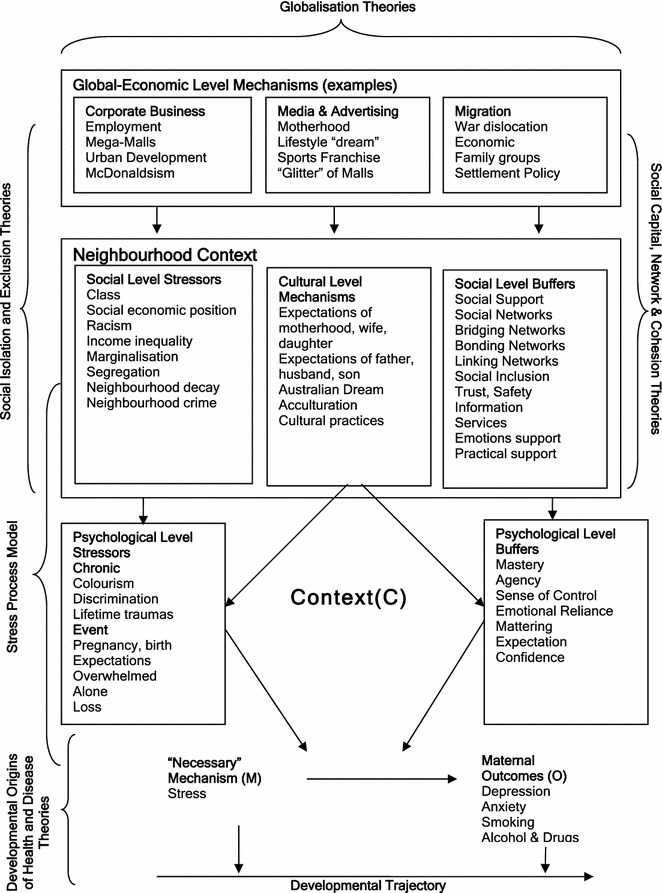
Conceptual Framework of Maternal Depression, Stress and Context

Drawing on the Theory Construction Analysis presented here a Conceptual Framework of Maternal Depression, Stress and Context was constructed (Fig. 8). That Conceptual Framework will form the basis for the development of a Middle Range Theory of Maternal Depression, Stress and Context.

Assessment of Inference to the Best Explanation for the Conceptual Framework of Maternal Depression, Stress and Context, using Hills “aspects of association” and Thagard’s principles and criteria, is shown (Table 7). In summary the Conceptual Framework of Maternal Depression, Stress and Context provides the best explanation of the data analysed.

**Table 7:** Conceptual framework of maternal depression, stress and context—inference to best explanation

Criteria	Application
*Hill’s aspects of association*	
Strength	Final models integrate strong associations of financial stress, lack of support, lost expectations and findings related to migrant mothers
Consistency	Final models consistent with earlier models and analysis
Specificity	No specificity identified
Temporality	No temporality demonstrated in this study
Biological gradient	Gradients were demonstrated in individual and ecological studies
Plausibility	The associations described are plausible
Coherence	The association is coherent with what is know
Experimental evidence	No experimental evidence was identified
Analogy	There are analogies between the propositions here and The Stress Process Model
*Thagard’s Principles*	
Symmetry	There is symmetry in the final models depression
Explanation	The propositions (a) coheres with evidence on depression, (b) coheres with other propositions and (c) are not a single proposition.
Analogy	There are analogies between the propositions here and The Stress Process Model
Data priority	The propositions describe the QUANT and QUAL data observations.
Contradiction	There are no contradictory proposals
Competition	No competitive explanation identified where p and q were not explanatorily connected
Acceptance	The propositions are coherent with the overall system of propositions
*Thagard’s Criteria*	
Consilience	The propositions explain a significant range of known facts
Simplicity	The propositions are the most simple set of explanations
Analogy	There are analogies between the propositions here and The Stress Process Model

### Methodological approaches

As previously noted in the protocol to this study (Eastwood et al. 2014) “we have used here the meta-theory of critical realism for the generation of causal explanations in social epidemiology as a response to the criticisms put forward by Muntaner (1999), O’Campo (2003) and Raphael (2006)”. The study demonstrates that critical realism can provide the necessary meta-theoretical philosophy for the generation of social epidemiology theory. The stratifying of reality demands that the researcher examines and explains unobserved generative forces (i.e., social isolation) that shape experiences. As previously observed “the fallibility of observations (and thus knowledge) is partly explained by the ontological separation of actual and observed realms together and the influence of context on the generative mechanism(s) an experienced phenomenon” (Eastwood et al. 2014a).

As argued and demonstrated in this study both qualitative and quantitative methods are able to contribute to the emergent, explanatory and confirmatory phases of theory building. Such a pluralist approach is embraced by critical realist methodologists through what Sayer (2000) calls intensive and extensive study designs respectively. From a philosophy of epidemiology perspective, Russo (2009) argues that evidence about both “difference-making” (c.f. regularities) and mechanisms is required for the development of causal inference”.

The concurrent triangulation design used here provided for strong integration with constant comparison across the concurrent studies and triangulation of findings. The study was able to demonstrate the benefits for epidemiology research of using triangulation to formulate explanation and propositions based on the empirical observations. The implication here is that mixed method research has an important role to play in future social epidemiology research.

As described in the study protocol (Eastwood et al. 2014a) we have incorporated here the “emergent and confirmatory theory building approaches within an overarching critical realist explanatory theory building framework. The resulting pluralistic and transdisciplinary Explanatory Theory Building Method has the potential to make a significant contribution to population health and social epidemiology theory building”(Eastwood et al. 2014a).

The Emergent Phase drew on qualitative emergent and grounded theory and quantitative exploratory data analysis traditions with their utility for theory generation. The Construction Phase made explicit the abstract analytical process of abduction and Inference to Best Explanation (IBE) from where hypothetico-deductive theory testing typically starts and emergent theory building finishes (Lynham 2002). The main study, and the theory construction reported here, has demonstrated that the emergent and construction phases of Explanatory Theory Building Method can be applied to the field of social epidemiology and population health theory building as they have previously in disability (Danermark and Gellerstedt 2004) and development research (Olsen 2001). Confirmatory approaches within a realist philosophy have been also been successfully demonstrated within social epidemiology (O’Campo et al. 2009) and health policy and programme evaluation (Greenhalgh et al. 2009).

### Implications for policy and programme development

This study has identified significant spatial disparities with depressed mothers living in suburbs with low social capital, low ethnic diversity, low average adult education, low family incomes and high density. Multilevel studies found that these were predominantly compositional effects mirroring the individual level findings. Thus depressed mothers with financial difficulties, poor social networks and a non-Australian background are living in poor neighbourhoods with low social capital.

While physical attributes of neighbourhoods may be important, this study identified mechanisms that are generated by social, cultural and global-economic level structures and generative mechanisms. The implications for policy and practice are broad as the stressor and buffering mechanisms might be triggered or constrained at a number of levels. For example, the global financial circumstances have had an impact on the Australian Government’s ability to implement maternity leave provision, and several large employers have recently moved their manufacturing to South East Asian economies. At the same time the corporate business entities will build large shopping malls new South West Sydney communities with possible impacts on local suburbs.

The long term consequences of perinatal depression indicate that a public health approach is justified. The qualitative findings from this study suggest that health worker home visiting and phone contact may be helpful. A recent comprehensive review by Dennis (2005), which included a number of nurse home visiting programmes, found that the provision of intensive professional postpartum support was the most promising. In addition, the efficacy of home visiting for postnatal depression has recently been confirmed (Morrell et al. 2009). Social support networks were protective in the study reported here suggesting that antenatal interventions that promote friendship groups may be beneficial. The role of antenatal groups in preventing postnatal depression, however, have not been confirmed (Austin 2003) but proactive telephone-based peer support may be protective (Dennis et al. 2009).

The findings from this study and recent intervention studies indicate that there is merit in maternal and child health services continuing to develop and evaluate interventions that provide early support for mothers who have, or are “at risk” of developing depression. As we have previously observed (Eastwood et al. 2012) “the study’s findings related to expectations also have implications for antenatal education and counselling interventions. It may be beneficial to provide more information on the rewards and challenges of early parenthood (Harwood et al. 2007)”.

It is our intention to use the conceptual framework constructed in this study to develop a perinatal child and family “middle range” theory and consequently a realist programme theory that can inform local implementation strategies. That analysis will draw on other relevant findings from this study including: the spatial distribution of depression and poor access to primary care services; the impact of strong social capital on minority groups; the importance of social services and local social policy; and the potential role of local government and corporate business initiatives.

## Conclusion

The purpose of this study was to construct a theory of maternal depression and neighbourhood context using a critical realist approach to explanatory theory building. The analysis commenced with an overview of the philosophical and methodological approach to the Explanatory Phase of Theory building. The approach used included definition of stratified levels, analytical resolution, abductive reasoning, comparative analysis of quantitative and qualitative findings, retroduction, postulate and proposition development, and finally the review and comparison of relevant theories.

A conceptual framework is described which includes examples of mechanisms at psychological, social, cultural and global-economic levels. Stress was identified as a necessary mechanism that has the tendency to cause several outcomes including depression, anxiety, and health harming behaviours. The conceptual framework utilised the stress process model as a starting point and included conditional mechanisms identified through retroduction.

## Additional files

10.1186/s40064-016-2729-9 Acculturation theory.
10.1186/s40064-016-2729-9 Review of other theoretical frameworks.

## Abbreviations

LGA: Local Government Area
EPDS: Edinburgh Postnatal Depression Scale
ABS: Australian Bureau of Statistics
OLS: ordinary least squares
BYM: Besag, York and Mollie’
pD: effective number of parameters in the model
DIC: deviance information criteria
RR: relative risk
EFA: exploratory factor analysis
IRSD: Index of Relative Social Deprivation
ICE: Index of Concentrated Extremes
NESP: non-English speaking
SWS: South Western Sydney
SWSAHS: South Western Sydney Area Health Service
NSW: New South Wales
IBIS: Ingleburn Baby Information System
PCA: principal component analysis
WBCA: women of child-bearing age
CAR: conditional auto-regression
MC: Monte Carlo
