# Supplementary material for: Realist theory construction for a mixed method multilevel study of neighbourhood context and postnatal depression
Source: Springerplus. 2016 Jul 15;5(1):1081. doi: 10.1186/s40064-016-2729-9 (PMC4945545; doi:10.1186/s40064-016-2729-9)
Supplement: Supplementary file 1 — 10.1186/s40064-016-2729-9 Acculturation theory. [file 40064_2016_2729_MOESM1_ESM.docx]

## Appendix 1: Acculturation Theory

### Introduction

The phenomenon of postnatal depression was associated at an individual and ecological level with mothers not being born in Australia and ethnic segregation respectively. A number of abstract or conceptual frameworks might be used to redescribe or recontexualise these findings including: racism, racial or ethnic segregation and diversity, cultural theory, migration and acculturation theory. Colonisation and waves of migration over 200 years provide a historical backdrop to this study. It is within this context that we have elected to ascribe meaning to these phenomena within an acculturation^[[1]](#footnote-1)^ conceptual framework.

We might have used segregation as a conceptual framework but considered Acculturation included segregation and would allow for a broader recontextualisation of the phenomena identified. The earlier discussion of stress, social exclusion, social isolation, and social capital are also relevant here.

An eco-cultural framework described by Berry and colleagues ([2002](#_ENREF_8)) attempted to link cultural and ecological factors to psychological outcomes. The authors described the antecedent mechanisms as related to population-level characteristics such as the ecological and socio-political context. The outcomes described were observable behaviours and psychological characteristics and the intervening processes were assumed to be genetic transmission, cultural transmission, ecological influences and acculturation ([Van de Vijver et al. 2008](#_ENREF_38)).

Acculturation has a broad range of meanings including the socialization of children to the norms of their own culture. Here we will use it to mean the modification of the culture of a group or individual as a result of contact with a different culture. A contribution from the field of acculturation research describes acculturation outcomes as including psychological well-being (e.g. distress and mood states), socio-cultural competence in ethnic culture and socio-cultural competence in the mainstream culture ([Arends-Toth et al. 2006](#_ENREF_3)) cited by ([Van de Vijver et al. 2008](#_ENREF_38)).

Nauck ([2008](#_ENREF_31)) observes that acculturation is by nature a multilevel issue and that the most widely accepted approach to acculturation in cross-cultural psychology is that of Berry ([1997](#_ENREF_7)). The model proposed four individual level outcomes of the acculturation process. They are: integration, assimilation, separation and marginalisation. Nauck ([2008](#_ENREF_31)) is critical of the model as a multilevel framework arguing that a ”full-fledged multilevel study of acculturation … should include group dynamics between immigrants and the population majority”. He then proposes that social contexts influence individual level behaviour through: social structures that provide opportunities and constraints, social control within social networks, places of cultural transmission, migration goals and context specific identification (e.g. within ethnically homogenous peer groups). Thus of direct relevance to understanding acculturation are ecological level studies of racial and ethnic integration or segregation as undertaken here.

### Comparative Analysis

#### Triangulation

The possibility of acculturation as a [generative mechanism] of the phenomenon of postnatal depression emerge from our group level qualitative study ([Eastwood et al. 2014c](#_ENREF_23)) and was supported by the findings of the logistic regression ([Eastwood et al. 2012a](#_ENREF_19)), principal component analysis ([Eastwood et al. 2012b](#_ENREF_21)) and ecological studies and multilevel studies ([Eastwood et al. 2014a](#_ENREF_16); [Eastwood et al. 2013a](#_ENREF_18); [Eastwood et al. 2013b](#_ENREF_20)). The interview guide was altered to integrate those quantitative findings and subsequently encouraged exploration of cultural and ethnic issues in the focus groups.

The qualitative interviews and focus groups reported a belief that maternal depression was higher among migrant families. This was confirmed by the quantitative studies ([Eastwood et al. 2012a](#_ENREF_19); [Eastwood et al. 2012b](#_ENREF_21)). In relation to unplanned pregnancy it was observed that where a mother was not married different cultures may respond in different ways. Those responses were not thought to be ethnically determined and it was noted that cultural differences also existed within mainstream society.

Practitioners interviewed identified a link between social cohesion and ethnic segregation. Divisions between Arabic and Vietnamese groups were noted with a tendency for large groups to become quite separate. By contrast in more diverse communities migrant women participated in multicultural groups because “we want to be part of our community”. By contrast in communities with large homogenous ethnic groups mothers did not want to meet with others because they didn’t like the way women from other cultures parented.

*“What I found startling was, what we find in Bankstown is there is a huge Vietnamese community and a huge Arabic community. Is it because they are so large that they end up becoming quite separate? Where as in the other two, Liverpool and Fairfield, does the salt and peppering of the multinational nature of it mean that there is a greater degree of integration?”*

The segregation may not be a problem until there is a pregnancy and infant. One expert explained it as follows

*“They might be self-sufficient. Working in a restaurant they are liaising with Vietnamese that is fine. But once you have a pregnancy they will have to go out to access services [and] be part of the broader society for schooling and for everything. That is why the isolation is highlighted because they don’t know the system. They might have been fully integrated into Cabramatta and this pregnancy has to bring them out of their comfort zone.”*

Also highlighted was the role that Health and Community services might be playing in creating segregation. One expert felt that services were so “fully into addressing the needs of particular religions, or specific groups, that we are creating segregation because it is an easy solution for us”. She gave the example of “Like say Arabic Muslim from Bankstown wouldn’t go to Lakemba. They are different. Although they are both Muslims they wouldn’t mix”.

Mothers in the focus groups also highlighted the possible role of ethnic clusters. One mother when asked why some women might get more depressed than others wondered if it may be because there are clusters of people from different cultures living in those communities. She said that she knew that depression was more prevalent in mothers of a certain ethnic group and therefore if there was a high concentration of those people in the suburb there would be more depression.

There appeared to be a contradiction in what would be best for mothers. One expert felt that if every Muslim women “who identify let’s say as Suni” was sent to a particular establishment then we would be creating a cluster or group that would not know anything except that. She felt it was a risk not to expose them to other groups.

*“So we are addressing this sense of belonging but then we are creating nuclease where this cluster of people, where they don’t know anything of the broader society”*

A different expert noted that people from similar ethnic groups seek each other out. Many of those ethnic communities are a “supportive family orientated group” where the women are very supportive of each other. They may come from large families where there are many sisters, aunties and grandmothers and the women often do not work.

*“So in terms of parenting and having babies, for women from some of those ethnic backgrounds, the Arabic and African groups, are very supportive and very helpful.”*

The concurrent group-level exploratory factor analysis and spatial exploratory data analysis suggested a possible interplay between social disadvantage, and ethnic integration or segregation. Indices of ethnic segregation were higher in those suburbs with social disadvantage. One expert had wondered about this relationship in the suburb of Miller.

*“I have often wondered in Miller the kind of interplay, racism is going up and people don’t think that multiculturalism makes life better. I’ve often wondered if that as an area becomes more depressed and disadvantaged and there’s less opportunities and lots of structural problems, and less connectiveness. Whether that means people will go at each more and won’t be able to support each other. Where as if you’ve got those other things in place, the ethnic diversity comes into play in a good way”.*

The individual level logistic regression study ([Eastwood et al. 2012a](#_ENREF_19)) confirmed our earlier South West Sydney findings of an association between mother not being born in Australia and postnatal depressive symptoms ([Eastwood 2005](#_ENREF_14)). The variable “country of birth” was also analysed using non-linear PCA ([Eastwood et al. 2012b](#_ENREF_21)) and CFE specification search ([Eastwood 2011, pp 175-77](#_ENREF_15)). In the non-linear PCA “country of birth” loaded most strongly on dimension 4 together with self-reported health, health of child, social support network and marital status. In the four factor measurement model specification search country of birth, social support network and practical support loaded on Factor 2 which we labelled “ethnic social marginalisation”.

The group level quantitative studies included a three of indicators of ethnic segregation and diversity – Entropy Segregation, Simpson Segregation and Maly Neighbourhood Diversity indices ([Eastwood et al. 2014b](#_ENREF_17)). In the exploratory factor analysis the Entropy Segregation Index loaded on Factor 2 together with low volunteerism, poor schooling, high density low class and low IRSD deciles. By contrast the Maly (Ethnic) Diversity Index loaded on Factor 6 with increased support network, increased practical support, better schooling, low density, and different address in the past 5 years.

Ecological visualisation and bivariate analysis found associations between measures of segregation and postnatal depression. The Maly Index of Diversity was weakly associated. In both the ecological likelihood linear and Bayesian spatial multiple regressions “Entropy” remained significant in the models for EDS >9 and EDS > 12 ([Eastwood et al. 2013b](#_ENREF_20); [Eastwood et al. 2013c](#_ENREF_22)).

The Bayesian multi-level spatial analysis found for EDS > 12 the density, the Maly Index and Factor 2 improved the model but were not significant. Low % no support, low % poor schooling and Factor 6 improved the model and were significant. Factor 6 significantly improved the Bayesian multi-level model reducing the DIC from 5215 to 5201 ([Eastwood 2011, pp 296-304](#_ENREF_15)).

In the stratified multilevel analysis measures of segregation and diversity did not improve the model for mothers born in Australia but for those mothers not born in Australia the Maly Index improved the model for EDS >9 and EDS > 12 ([Eastwood et al. 2013b](#_ENREF_20)).

The ecological studies (linear regression and spatial) found a strong association of aggregated postnatal depression with aggregated measures segregation. When controlling for individual level factors (including not born in Australia) the multilevel measures of segregation were no longer significant. In the stratified study of mothers not born in Australia there was a group level protective effect from increased neighbourhood ethnic diversity (Maly Index). The concomitant paradoxical association with low aggregated social networks is discussed on the main manuscript in relation to Social Capital Theory.

The quantitative findings suggest that ethnic segregation is operating predominantly at the individual family level. By contrast group level ethnic diversity is protective for mothers not born in Australia living in communities with high levels of social capital (social networks).

Recontextualisation of these multilevel findings within the acculturation conceptual framework suggests that for migrant mothers, social relations (and possible bridging networks) are stronger in ethnically diverse communities and protect against maternal depression. The converse logic is that, for migrant mothers, there is more social support than might be expected, in suburbs with low aggregated (ecological) social support.

#### Ethnicity and Perinatal Depression

Perinatal depression has been found to be more common among recent migrants to Australia ([Brown and Lumley 2000](#_ENREF_10); [Brown et al. 1994](#_ENREF_11); [Lansakara et al. 2010](#_ENREF_26); [Williams and Carmichael 1985](#_ENREF_39)). Small and colleagues found that the rates were high among Turkish women but relatively low among Vietnamese and Filipino women([Small et al. 2003](#_ENREF_34)). Rates of postnatal depression among migrants have also been found to be high among Pacific Island mothers in Auckland, New Zealand ([Abbott and Williams 2006](#_ENREF_1)), Canadian immigrants, asylum seekers and refugees ([Dennis et al. 2009](#_ENREF_12); [Stewart et al. 2008](#_ENREF_35); [Sword et al. 2006](#_ENREF_37)), London ethnic minorities ([Onozawa et al. 2003](#_ENREF_32)) and Latinas or Hispanic US mothers ([Beck et al. 2005](#_ENREF_5); [Diaz et al. 2007](#_ENREF_13)).

The significance of these findings is complicated by the wide international cross-cultural variation in postnatal depression and depressive symptoms. Halbreich and Karkum undertook a review of 143 studies from 40 countries and found a wide range in reported rates ([2006](#_ENREF_24)). The authors concluded that the variability might be due to cross-cultural variables, reporting style, differences in perception of mental health and its stigma, differences in socio-economic environments and biological vulnerability factors.

Bina ([2008](#_ENREF_9)) analysed 70 studies on culture and postnatal depression. Of those she explored in depth 14 studies that focused on the impact of cultural factors on postnatal depression. Researchers in 8 of those studies concluded that cultural rituals alleviated postnatal depression and that a lack of cultural traditions leads to increased rates of depression. The most common ritual identified by Bina was resting for a period after birth with support from the extended family (usually the mother or mother in law). Social support was again identified as important but specifically the review highlighted the importance of the woman’s perception of support ([Bina 2008](#_ENREF_9)).

Of direct relevant to this study are the findings of Stuchbery and colleagues ([1998](#_ENREF_36)) who undertook a study of Vietnamese, Arabic and Anglo-Celtic mothers in South West Sydney specifically to examine which deficits in their social support network were associated with postnatal depression among mother of a non-English speaking background. In summary, for Anglo-Celtic women, low postnatal mood was associated with a perceived need for more emotional support from partners and mothers. For Vietnamese low mood was associated with poor quality relationship with the partner and a perceived need for more practical support from him. For Arabic women low mood was associated with a perceived need for more emotional support from partners. The authors noted the importance of postnatal rituals. In their study 64 percent of Vietnamese and 61 percent of Arabic women did not have their mothers with them.

Beck cited Nahas and Amasheh ([1999](#_ENREF_30)) who had explored the experiences of postnatal depression among Jordanian women living in Australia. Nahas and Amasheh had found that “Jordanian women were not supposed to be sad because in their culture this means that the women are not at all able to cope and are bad mothers. Living in a country that was not their homeland only accentuated these conflicts. Jordanian mothers living in Australia did not have strong family support they were used to in their own country. These depressed women experienced helplessness due to their inability to fulfil their traditional gender roles as wife and mother” (([Beck 2002](#_ENREF_6)) citing ([Nahas and Amasheh 1999](#_ENREF_30))).

It is clear from this brief overview of literature on ethnic migration and postnatal depression or depressive symptoms that the elements of stress and support continue to play an important role. There is clearly a cultural context to both the perception and reality of these two important elements.

#### Segregation and Integration Literature

An important subset of research into neighbourhood effects is the study of racial, ethnic, and socioeconomic segregation. This is where research investigates whether the observed differences in outcomes are attributable to the fact that different sub-groups live in different social, physical and institutional environments ([Reardon 2006](#_ENREF_33)).

There is an extensive literature concerning racial segregation principally from the United States where African Americans are significantly more segregated that the white population or other racial/ethnic minorities. The effects of segregation on health have been thus examined principally in relation to the health status of US Blacks ([Acevedo-Garcia and Lochner 2003](#_ENREF_2); [Kramer and Hogue 2009](#_ENREF_25)). In their review of 15 such studies Acevedo-Garcia and Lochner ([2003](#_ENREF_2)) authors found that no studies explored explanations of why and how residential segregation influences health, the testing of specific pathways and the development of multilevel models. They hypothesized that segregation had an indirect effect on health outcomes operating through multiple mediator variables ([Acevedo-Garcia and Lochner 2003](#_ENREF_2)).

Kramer and Hogue identified 39 studies that tested an association between segregation and health outcomes. The health effects were described as complex with isolation segregation associated with poor pregnancy outcomes and increased mortality for blacks. Several studies, however, reported health-protective effects of living in clustered black neighbourhood after controlling for social and economic isolation.

The majority of the segregation measures developed have focused on analysis of residential separation of whites and blacks in the US. Maly ([2000](#_ENREF_27)) developed the Neighbourhood Diversity index which allows researchers to include multiple racial and ethnic categories while adhering to a focus on integration rather than segregation. These measures of segregation and integration have allowed researchers to study urban residential changes in ethnic integration both spatially and over time ([McCulloch 2007](#_ENREF_28); [Modarres 2004](#_ENREF_29)). The importance of using measures of both segregation and integration are illustrated below.

Ethnic segregation may provide health advantages or conversely be detrimental. Consistent with this notion, in a small study of a disadvantaged minority community in Alabama, Mitchell and LaGory (2002) reported that high bonding social capital (measured by the strength of trust and associational ties with others of a similar racial and educational background as the respondent) was paradoxically associated with higher levels of mental distress. In the same study, however, individuals who reported social ties to others who were unlike them with respect to race and class (i.e. who had access to bridging capital) were less likely to report mental distress.

To recontexualise this study within an Acculturation framework provides an alternative theoretical perspective. The four individual level outcomes of the acculturation process are: integration, assimilation, separation and marginalisation. Within the Acculturation framework “bridging capital” is also “integration” and provides positive mental health benefits whereas “high bonding capital” is “segregation” and is associated with higher levels of mental distress.

#### Abductive and Retroduction Analysis

Redescribing ethnicity and perinatal depression within an acculturation conceptual framework provides explanation for a number of possible causative mechanisms including cultural traditions of postnatal support, marginalisation, societal prejudice and discrimination, social support, ethnic integration and segregation. Specific to this conceptual framework is a possible causative mechanism called acculturation. A recent study by Ayers and colleagues ([2009](#_ENREF_4)) found that a composite measure of acculturation, among US Korean immigrant women, had an indirect protective relationship with depression mediated through immigration stress. The mechanism of acculturation was not associated with social support which, in their study, had a direct protective effect on depression.

Structures within the global, political and social levels that may generate conditional causative mechanism include: global markets, big business, political structures, dominant culture, government agencies, religious organisations, and civil society structures. Candidate conditional mechanisms may include: immigration and refugee policy, skills shortages, societal prejudice and discrimination, religious tolerance, and social cohesion.

As noted above, recontextualisation of paradoxical multilevel findings within the acculturation conceptual framework suggests that for migrant mothers, social relations (and possibly bridging networks) are stronger in working class communities and protect against maternal depression. The converse logic was that, for migrant mothers, there is more social exclusion than might be expected, in suburbs with extremes between rich and poor.

We have examined the related causal and conditional mechanisms of stress, social isolation, social exclusion, social support networks and social services. We propose that the following conditional mechanisms may influence the tendency of acculturation to reduce social isolation, stress and depression:

1. Immigration policy (i.e. family members, promote tolerance)
2. Media policy and response toward migration and refugees
3. Global market and business approach to migration
4. Civil society response to migration
5. Settlement patterns (i.e. integration or segregation)
6. Linking social capital and social cohesion
7. Strong ethnic bonding networks
8. Strong bridging networks
9. Access to services including home visiting nursing services.

### Proposition

A critical realist model of the ethnic migrant mechanisms is below.

Ethnic Migrant Mechanisms

Acculturation

Cultural Practices

Integration

Conditions (other mechanisms)

- .Immigration policy
- Media policy and response
- Global market and business approach
- Civil society response to migration
- Settlement patterns
- Linking social capital and social cohesion
- Strong ethnic bonding networks
- Strong bridging networks
- Access to services.

Reduced Stress

Increased support

Outcomes

Figure A1: Critical Realist Model of Acculturation & Ethnic Integration Propositions

Based on the above analysis the following propositions are made:

- Immigrant acculturation is a social level mechanism that protects against maternal stress when certain personal characteristics and contextual conditions exist.
- Ethnic segregation is a social level mechanism that increases maternal isolation when certain personal characteristics and contextual conditions exist
- Ethnic integration is a social level mechanism that increases maternal support when certain personal characteristics and contextual conditions exist.

Assessment of Inference to the Best Explanation for the above propositions, using Hills “aspects of association” and Thagard’s principles and criteria, is shown below.

| **Criteria** | **Application** |
| --- | --- |
| ***Hill’s aspects of association*** | |
| Strength | Acculturation and ethnic mechanisms were identified by QUAL and QUANT as associated with depression at the individual and ecological levels. |
| Consistency | Ethnic and migrant factors have been previously identified as associated with maternal depression |
| Specificity | No specificity identified |
| Temporality | No temporality demonstrated in this study |
| Biological gradient | There was a gradient at the ecological level |
| Plausibility | The association between migration and ethnic segregation with stress is plausible |
| Coherence | The association is coherent with what is know |
| Experimental evidence | No experimental evidence was identified |
| Analogy | There is an analogy between of ethnic segregation and social exclusion both causing stress |
| ***Thagard’s Principles*** | |
| Symmetry | There is symmetry between ethnic segregation causing stress and the buffering of stress by social support and practical support thus preventing depression |
| Explanation | The ethnic and acculturation propositions a) coheres with evidence on depression, b) coheres with other propositions and c) is not a single proposition. |
| Analogy | There is an analogy between of ethnic segregation and social exclusion |
| Data priority | The proposition describes the data observations. |
| Contradiction | There are no contradictory proposals |
| Competition | No competitive explanation identified where p and q were not explanatorily connected |
| Acceptance | The ethnic and acculturation propositions are coherent with the overall system of propositions |
| ***Thagard’s Criteria*** | |
| Consilience | Ethnic and acculturation propositions explains a limited range of known facts |
| Simplicity | Ethnic and acculturation propositions are not sufficient to explain depression. Not the most simple explanation |
| Analogy | There is an analogy between of ethnic segregation and social exclusion |

Table A1: Acculturation proposition – Inference to Best Explanation

In summary the Ethnic and acculturation propositions are not the best explanation. It explains some of the observed and known evidence but is insufficient on its own.

### References

Abbott MW, Williams MM (2006) Postnatal depressive symptoms among Pacific mothers in Auckland: Prevalence and risk factors. Aust N Z J Psychiatry 40(3):230-238

Acevedo-Garcia D, Lochner K (2003) Residential Segregation and Health. In: Kawachi I, Berkman L (eds) Neighborhoods and Health. Oxford University Press, Oxford, UK

Arends-Toth J, Van de Vijver F, Poortinga Y (2006) The influence of method factors on the relation between attitudes and self-reported behaviors in the assessment of acculturation. European Journal of Psychological Assessment 22

Ayers J, Hofstetter R, Usita P, Irvin V, Kang S, Hovell M (2009) Sorting out t competing effects of acculturation, immigrant stress, and social support in depression: A report on Korean Women in California. The Journal of Nervous and Mental Disease 197(10):742-747

Beck C, Froman R, Bernal H (2005) Acculturation level and postpartum depression in Hispanic mothers. MCN Am J Matern Child Nurs 30(5):299

Beck CT (2002) Postnatal Depression: A Metasynthesis. Qual Health Res 12(4):453-472

Berry J (1997) Immigration, acculturation, and adaptation. Applied Pscyhology: An International Review 46:5-34

Berry J, Poortinga Y, Segall M, Dasen P (2002) Cross-cultural psychology: Research and applications (2nd ed.). Cambridge University Press, Cambridge

Bina R (2008) The Impact of Cultural Factors Upon Postpartum Depression: a Literature Review. Health Care for Women International 29:568-592

Brown S, Lumley J (2000) Physical health problems after childbirth and maternal depression at six to seven months postpartum. International Journal of Obstetrics and Gynaecology 107(10):1194-201

Brown S, Lumley J, Small R, Astbury J (1994) Missing voices: The experience of motherhood. Oxford University Press, New York

Dennis C, et al. (2009) Effect of peer support on prevention of postnatal depression among high risk women: multisite randomised controlled trial. Br Med J 338:a3064

Diaz M, Le H, Cooper B, Muñoz R (2007) Interpersonal factors and perinatal depressive symptomatology in a low-income Latina sample. Cultur Divers Ethnic Minor Psychol 13(4):328

Eastwood J (2005) Postpartum Depression: A Population Based Study. Masters, University of New South Wales

Eastwood J (2011) Realist Theory Building for Social Epidemiology: Building a Theoretical Model of Neighbourhood Context and the Developmental Origins of Health and Disease Using Postnatal Depression as a Case Study. PhD Thesis, University of New South Wales. Available at: <http://handle.unsw.edu.au/1959.4/50896>

Eastwood J, Jalaludin B, Kemp B, Phung H (2014a) Bayesian Hierarchical Spatial Regression of maternal depressive symptoms in South Western Sydney, Australia. SpringerPlus 3:55 doi:10.1186/2193-1801-3-55

Eastwood J, Jalaludin B, Kemp B, Phung H (2014b) Realist identification of group-level latent variables for perinatal social epidemiology theory building. Int J Health Serv 44(3):407-433

Eastwood J, Jalaludin B, Kemp L, Phung H, Adusumilli S (2013a) Clusters of maternal depressive symptoms in South Western Sydney, Australia. Spat Spatiotemporal Epidemiol 4:25-31

Eastwood J, Jalaludin B, Kemp L, Phung H, Barnett B (2012a) Relationship of Postnatal Depressive Symptoms to Infant Temperament, Maternal Exepectations, Social Support and Other Potential Risk Factors: Findings from a large Australian Cross-Sectional Study. BMC Pregnancy Childbirth 12:148

Eastwood J, Jalaludin B, Kemp L, Phung H, Barnett B (2013b) Immigrant maternal depression and social networks. A multilevel Bayesian spatial logistic regression in South Western Sydney, Australia. Spat Spatiotemporal Epidemiol 6:49-58

Eastwood J, Jalaludin B, Kemp L, Phung H, Barnett B, Tobin J (2012b) Social exclusion, infant behaviour, social isolation and maternal expectations independently predict maternal depressive symptoms. Brain and Behavior 3:14-23

Eastwood J, Jalaludin BB, Kemp L, Phung H (2013c) Neighbourhood adversity, ethnic diversity, and weak social cohesion and social networks predict high rates of maternal depressive symptoms: A critical realist ecological study in South Western Sydney, Australia. Int J Health Serv 43(2):241-266

Eastwood J, Kemp L, Jalaludin B (2014c) Explaining ecological clusters of maternal depression in South Western Sydney. BMC Pregnancy Childbirth 14:47 doi:10.1186/1471-2393-14-47

Halbreich U, Karkun S (2006) Cross-cultural and social diversity of prevalence of postpartum depression and depressive symptoms. J Affect Disord 91(2-3):97-111

Kramer M, Hogue C (2009) Is Segregation Bad for Your Health. Epidemiol Rev 31:178-194

Lansakara N, Brown S, Gartland D (2010) Birth Outcomes, Postpartum Health and Primary Care Contacts of Immigrant Mothers in an Australian Nulliparous Pregnancy Cohort Study. Matern Child Health J 14(5):807-16 doi:10.1007/s10995-009-0514-x

Maly M (2000) The Neighborhood Diversity Index: A Complementary Measure of Racial Residential Settlement. J Urban Aff 22(1):37-47

McCulloch A (2007) The changing structure of ethnic diversity and segregation in England, 1991-2001. Environment and Planning A 39:909-927

Modarres A (2004) Neighbourhood Integration: Temporality and Social Fracture. J Urban Aff 26(3):351-377

Nahas V, Amasheh N (1999) Culture care meanings and experiences of postpartum depression among Jordanian Australian women: A Transcultural study. J Transcult Nurs 10:37-45

Nauck B (2008) Acculturation. In: Van de Vijver F, Van Hemert D, Poortinga Y (eds) Multilevel Analysis of Individuals and Cultures. Lawrence Erlbaum Associates, New York

Onozawa K, Kumar R, Adams D, Dore C, Glover V (2003) High EPDS scores in women from ethnic minorities living in London. Arch Womens Ment Health 6(Suppl2):s51-s55

Reardon S (2006) A conceptual framework for measuring segregation and its association with population outcomes. In: Oakes J, Kaufman J (eds) Methods in Social Epidemiology. Jossey-Bass, San Francisco, CA

Small R, Lumley J, Yelland J (2003) Cross-cultural Experiences of Maternal Depression: Associations and Contributing Factors for Vietnamese, Turkish and Filipino Immigrant Women in Victoria, Australia. Ethn Health 8(3):189-206

Stewart D, Gagnon A, Saucier J, Wahoush O, Dougherty G (2008) Postpartum depression symptoms in newcomers. Can J Psychiatry 53(2):121-124

Stuchbery M, Matthey S, Barnett B (1998) Postnatal depression and social supports in Vietnamese, Arabic and Anglo-Celtic mothers. Soc Psychiatry Psychiatr Epidemiol 33:483-490

Sword W, Watt S, Krueger P (2006) Postpartum health, service needs, and access to care experiences of immigrant and Canadian-born women. Journal of Obstetric, Gynecologic and Neonatal Nursing 35:717-727

Van de Vijver F, Van Hemert D, Poortinga Y (2008) Conceptual Issues in Multilevel Models. In: Van de Vijver F, Van Hemert D, Poortinga Y (eds) Multilevel Analysis of Individuals and Cultures. Lawrence Erlbaum Associates, New York

Williams H, Carmichael A (1985) Depression in mothers in a multi-ethnic urban industrial municipality in Melbourne. Aetiological factors and effects on infants and preschool children. J Child Psychol Psychiatry 27:277-288

1. Note: we are using “Acculturation” here as a conceptual or theoretical framework for recontextualisation of phenomena described. We are not analysing the process “acculturation” which may, or may not be associated with postnatal depression. [↑](#footnote-ref-1)
