# Supplementary material for: Realist theory construction for a mixed method multilevel study of neighbourhood context and postnatal depression
Source: Springerplus. 2016 Jul 15;5(1):1081. doi: 10.1186/s40064-016-2729-9 (PMC4945545; doi:10.1186/s40064-016-2729-9)
Supplement: Supplementary file 2 — 10.1186/s40064-016-2729-9 Review of other theoretical frameworks. [file 40064_2016_2729_MOESM2_ESM.docx]

## Appendix 2: Review of other Theoretical Frameworks

The theoretical and conceptual frameworks selected for the analysis, in this Realist Theory Construction, were chosen after considering other relevant social epidemiological, social, community psychology, public health, perinatal and postnatal depression theories. Some of these are briefly discussed here.

### “Black box” Debate

Cognisance was given to the “black box debate” of the 1990s and the proposal for a new era called “eco-epidemiology” in which broad explanatory theories of disease and health in populations were developed consistent with a systems approach ([Pearce 1996](#_ENREF_27), [Susser and Susser 1996](#_ENREF_30), [Krieger 2001](#_ENREF_20)). The debate concerning the merits of “eco-epidemiology” versus “risk-factor (black-box) epidemiology continues ([Greenland, Gago-Dominguez et al. 2004](#_ENREF_15), [Susser 2004](#_ENREF_29)). Underlying these perspectives are epistemological questions of scientific theory ([Haack 2004](#_ENREF_16), [Mayo and Spanos 2004](#_ENREF_24)) including the discourse between inductivism, and deductive theory/refutationism.

### Eco-social Theory

In relation to the theoretical basis of social epidemiology we reviewed the theories explored by Krieger ([2001](#_ENREF_20)). Krieger critiqued three main theories used by practising social epidemiologists at that time namely: (1) psychosocial, (2) social production of disease and/or political economy of health, and (3) eco-social theory and related multi-level frameworks. Drawing on her earlier work ([1994](#_ENREF_18), [2000](#_ENREF_19)) Krieger argued for an eco-social construct that minimally included:

1. **“embodiment**, a concept referring to how we literally incorporate, biologically, the material and social world in which we live, from conception to death; a corollary is that no aspect of our biology can be understood absent knowledge of history and individual and societal ways of living.
2. **pathways of embodiment**, structured simultaneously by: (a) societal arrangements of power and property and contingent patterns of production, consumption, and reproduction, and (b) constraints and possibilities of our biology, as shaped by our species' evolutionary history, our ecological context, and individual histories, that is, trajectories of biological and social development;
3. **cumulative interplay between exposure, susceptibility and resistance**, expressed in pathways of embodiment, with each factor and its distribution conceptualized at multiple levels (individual, neighbourhood, regional or political jurisdiction, national, inter- or supra-national) and in multiple domains(e.g. home, work, school, other public settings), in relation to relevant ecological niches, and manifested in processes at multiple scales of time and space;
4. **accountability and agency**, expressed in pathways of and knowledge about embodiment, in relation to institutions (government, business and public sector), households and individuals, and also to accountability and agency of epidemiologists and other scientists for theories used and ignored to explain social inequalities in health; a corollary is that, given likely complementary causal explanations at different scales and levels, epidemiological studies should explicitly name and consider the benefits and limitations of their particular scale and level of analysis.”

### Health Theories of Inequality

Also considered were theories used to explain health inequalities. Bartley

([2004](#_ENREF_3)) discussed four of these*.* They were the: 1) Behavioural and Cultural, 2) Psychosocial, 3) Materialistic, and 4)Life-course theories.

The **Life-course theory** has been used in this thesis to argue for the importance of early influences on the foetus and infant. Life-course theory was incorporated as appropriate into the development of theory and conceptual models.

The **Psychosocial Theory** is central to the development of theories in this study but we have elected to focus the theory development process on two of its key elements namely stress and support. Krieger noted that the psychosocial theory “directs attention to endogenous biological responses of human interactions [and] focus is on responses to ‘stress’ and on stressed people in need of psychosocial resources. Comparatively less attention, theoretically and empirically, is accorded to: (1) who and what generates psychosocial insults and buffers, and (2) how their distribution – along with that of ubiquitous or non-ubiquitous pathogenic physical, chemical or biological agents – is shaped by social, political and economic policies” ([Krieger 2001](#_ENREF_20)). The psychosocial theory is central to this analysis and is integrated into the theoretical framework constructed.

**The Behavioural and Cultural theories** discussed by Bartley were principally related to life style behaviours which would be of direct relevance to consideration of perinatal tobacco and alcohol use. Of more relevance to this study was consideration of theories related to ethnic and cultural segregation, isolation and integration which were explored above.

**The Materialist and Neo-material Theories** relate to “evidence for the existence of material causes of health and inequality … which show that health is worse and life expectancy lower in people who have, or may reasonably be assumed to have, relatively low incomes “([Bartley 2004](#_ENREF_3)). Recently “neo-materialist” theory has emerged which concentrates on public provision, or subsidisation of services and utilities such as education, housing, water and transport. The ‘Materialist’ and ‘Neo-materialist’ Theories, with their elements of essential needs, social participation and public provision of subsidies and services have not been sued in this study. We elected to use the closely linked theory of social exclusion.

### Community Psychology Theories

The field of community psychology had much to offer the development of a theoretical approach to neighbourhood context and perinatal depression. Theoretical positions reviewed included: 1) Transactional and Ecological Theory, 2) Empowerment Theory, 3) Stress Theory, 4) Social Support Theory, and 5) Citizen participation.

**Transactional theory** is a theory that emphasises the dynamic, reciprocal interactions between the infant and their context, with bidirectional influence being a fundamental element. A comprehensive **ecological model of development** ([Bronfenbrenner 1979](#_ENREF_7)) has been linked to this transaction theoretical view. The resulting transactional – ecological (T-E) theory is similar to the eco-epidemiological and Eco-social theories discussed earlier and supports the general approach being taken in this Study.

**Empowerment Theory** can be considered as both a “value orientation for working in the community and as a theoretical concept for understanding the process and consequences of efforts to exert control and influence over decisions that affect one’s life, organisational functioning, and the quality of community life” ([Zimmerman 2000](#_ENREF_34)). Empowerment theory is understood at multiple levels including individual, organisational and community levels.

In his critical analysis of empowerment theory Zimmerman notes that it is difficult to measure empowerment and thus some have dismissed its usefulness. Zimmerman further notes that empowerment may be considered as equivalent to power and be linked to issues regarding the struggle for power, power relationships and efforts to exert control over, or influence community power structures ([Zimmerman 2000](#_ENREF_34)). Empowerment theory has significant heuristic application to the development of theory in relation to neighbourhood context and perinatal depression. There is, however, difficulty in measuring empowerment at the various levels will described by Zimmerman.

**Stress Theory** forms a central tenant of **Psychosocial Theory** and was used in the Theory Construction.

**Social Support Theory** forms a central tenant of **Psychosocial Theory, Stress Process Theory,** and **Social Capital Theory.** Barrera ([1986](#_ENREF_2)) distinguishes between the concepts of social embeddedness, enacted support, and perceived social support. According to Barrera “social embeddedness” refers to the characteristics of the individual’s social network. “Enacted support” refers to the actual helping behaviours that are exchanged. Six different kinds of helping transactions are proposed. They are: advice and information, emotional support, physical assistance, recreation, and positive feedback. **“Perceived support”** refers to the individual’s evaluation of the quality of support provided by the social network, that is, the degree to which it is seen as available and helpful.

**Citizen Participation** Theory concerns the “process in which individuals take part in decision making in the institutions, programs, and environments that affect them” ([Heller, Price et al. 1984](#_ENREF_17)) cited by Wandersman and Florin ([2000](#_ENREF_32)). Community participation is an important component of social cohesion.

### Theoretical Models of Neighbourhood Effects

Drawing on the work of Green and Ottoson ([1999](#_ENREF_14)) and Wandersman and Nation ([1998](#_ENREF_33)), Ellen and colleagues ([2001](#_ENREF_12)) propose that neighbourhoods can influence health outcomes through four pathways: (1) neighbourhood institutions and resources (2) stresses in the physical environment (3) stresses in the social environment and (4) neighbourhood based network and norms.

1. **Neighbourhood institutions and resources:** Neighbourhoods clearly differ in their resources such as parks, libraries, access to healthy food, public transportation, access to health care facilities and so on. Thus, the distribution of those institutionalized resources will have consequence for maternal and infant health outcomes. This pathway suggests that collective investment in the quality and quantity of social and material resources would contribute to the outcomes of individual children.
2. **Physical stresses in the neighbourhood environment:** the most commonly discussed way in which neighbourhoods influence health is through the proximity of polluting factories and toxic waste sites, which may increase people’s chance of contracting cancer and other illness. Aging and poorly maintained environments – crumbling sidewalks, decaying stairwells, and dangerous playgrounds – are likely to increase the risk of accidents. These conditions are more likely to affect families living in low income neighbourhoods.
3. **Social stresses in the neighbourhood environment:** people’s health status can be directly affected by the social conditions in a neighbourhood. For instance, living in a neighbourhood with high rates of crime, a mother is more likely to be injured. Furthermore, there have been evidences that exposure to social conditions such as crime, violence, and noise can lead to a higher level of stress. Elevated level of stress in turn may result in many diseases and unhealthy behaviours like smoking.
4. **Neighbourhood based social networks:** neighbourhood social networks may shape health outcomes through transmitting norms about accepted behaviour, communicating important information or providing social support. For instance, smoking or eating a high fat diet may be more socially acceptable in some neighbourhoods than in other or feeling of hopelessness and isolation are more widely spread among residents of poorer and less empowered communities.

Macintyre and colleagues ([1993](#_ENREF_22)) developed a framework that proposed that the following aspects of neighbourhoods might be health promoting or health damaging:

1. “Physical features of the environment shared by all residents in a locality (for example, air and water quality)
2. Availability of healthy environments at home, work, and play (for example, decent housing, secure and nonhazardous employment, safe play areas for children)
3. Services provided to support people in their daily lives (for example, education, transportation, street cleaning and lighting, and policing)
4. The socio-cultural features of a locality (for example, the political, economic, ethnic, and religious history and the degree of community integration
5. The reputation of an area (for example, how the area is perceived by residents, service or amenity planners, and investors) ([Macintyre, Maciver et al. 1993](#_ENREF_22))”.

([Macintyre and Ellaway 2003, p33](#_ENREF_21))

### Perinatal Models

A framework proposed by Misra et al ([2003](#_ENREF_25)) marries a life course perspective, incorporating forces that influence the health of women through successive stages of their lives and their repro­ductive cycles with a multiple determinants model. The perinatal health framework is an adaptation of the Evans and Stoddart ([1990](#_ENREF_13)) model of health determinants, which while acknowledging the direct influence that biological, behavioural, environmental, and social factors have on health status, provides a framework for understanding the interrelations between such factors.

The framework proposed by Misra et al ([2003](#_ENREF_25)) is focused on the perinatal period with an emphasis on how factors relate to the preconception and interconception periods, and how multiple factors interact to influence perinatal outcomes.

Culhane and Elo ([2005](#_ENREF_10)), reviewed the potential mechanisms through which neighbourhood context may influence perinatal outcomes. They outlined a conceptual framework that links neighbourhood context to adverse perinatal events highlighting important intervening variables along this pathway.

In the framework proposed by Culhane and Elo the neighbourhood conditions that are hypothesized to influence health, either directly or indirectly, are features of the neighbourhood’s social environment, service environment, and physical characteristics ([Robert 1999](#_ENREF_28)). Social environment are referred to as the level of neighbourhood cohesion or disorganization, norms of reciprocity, civic participation, crime, socioeconomic composition, residential stability, and related attributes. These characteristics are hypothesised to influence health outcomes through a number of potential pathways that include availability of social support, adaptation of coping strategies, and exposure to chronic stress ([Anderson, Sorlie et al. 1996](#_ENREF_1), [Diez Roux, Nieto et al. 1997](#_ENREF_11), [Taylor and Repetti 1997](#_ENREF_31), [Cubbin, LeClere et al. 2000](#_ENREF_9), [Buka, Brennan et al. 2003](#_ENREF_8), [Morenoff 2003](#_ENREF_26)).

Matthews and Meaney ([2005](#_ENREF_23)) provide an extensive review of the scientific basis for the influence of adverse perinatal events on life course outcomes. They noted that adversity associated with poverty produces incomplete, dysfunctional families often rife with domestic violence, drug use, child abuse and neglect and that reproduction within this context results in maternal stress, increased risks for infection and thus preterm labour, perinatal deaths, birth insults, poor nutrition for mother and offspring and serious compromises in the quality of parent-child interactions.

Matthews and Meaney ([2005](#_ENREF_23)) also noted that environmental adversity can compromise the emotional well-being of the parent and thus influence the quality of parent-child relationships and that that relationship is strongly linked to maternal anxiety and depression.

### Qualitative Studies of Postnatal Depression

Beck has undertaken extensive previous qualitative research on postnatal depression including a metasynthesis ([Beck 1992](#_ENREF_4), [Beck 1993](#_ENREF_5), [Beck 2002](#_ENREF_6)). Her phenomenological study of postpartum depression identified 11 themes that “described the essence of this experience”. Those “themes dealt with unbearable loneliness, obsessive thoughts, loss of self, suffocating guilt, cognitive impairment, loss of previous interests and goals, uncontrollable anxiety, insecurity, loss of control of emotions, loss of all positive emotions, and contemplation of death” ([Beck 1992](#_ENREF_4)).

Beck went on to develop a substantive theory of postpartum depression entitled “Teetering on the Edge” ([Beck 1993](#_ENREF_5)). Beck found that “Loss of control emerged as the basic social psychological problem”. She described women with postpartum depression as trying to “cope with this problem through the following four-stage process: (a) encountering terror, (b) dying of self, (c) struggling to survive, and (d) regaining control”.

In 2002 Beck published a metasynthesis of 18 qualitative studies on postpartum depression. Four overarching themes emerged from her metasynthesis that reflected four perspectives of postnatal depression: “(a) incongruity between expectations and the reality of motherhood, (b) spiralling downward, (c) pervasive loss, and (d) making gains ([Beck 2002](#_ENREF_6)).

we integrated this substantial body of previous qualitative research into the individual level qualitative theory generation (Section 4.6) and will utilise again here as part of the theory generation process.

### References

Anderson, R., P. Sorlie, E. Backlund, N. Johnson and G. Kaplan (1996). "Mortality effects of community socioeconomic status." Epidemiology **8**: 42-47.

Barrera, M. (1986). "Distinctions between social support concepts, measures and models." American Journal of Community Psychology **14**: 413-445.

Bartley, M. (2004). Health Inequality: An Introduction to Theories, Concepts and Methods. Cambridge, UK, Polity.

Beck, C. T. (1992). "The Lived Experience of Postpartum Depression: A Phenomenological Study." Nursing Research **41**(3): 166-170.

Beck, C. T. (1993). "Teetering on the edge: A substantive theory of postpartum depression." Nursing Research **42**(1): 42-48.

Beck, C. T. (2002). "Postnatal Depression: A Metasynthesis." Qualitative Health Research **12**(4): 453-472.

Bronfenbrenner, U. (1979). The ecology of human development: Experiments by nature and design. Cambridge, MA, Harvard University Press.

Buka, S., R. Brennan, J. W. Rich-Edwards, S. Raudenbush and F. Earls (2003). "Neighbourhood Support and the Birth Weight of Urban Infants." American Journal of Epidemiology **157**(1): 1-8.

Cubbin, C., F. LeClere and G. Smith (2000). "Socioeconomic status and injury mortality: individual and neighborhood determinants." Journal of Epidemiology & Community Health **54**: 517-552.

Culhane, J. and I. Elo (2005). "Neighbourhood context and reproductive health." American Journal of Obstetrics & Gynecology **192**(5 Suppl): s22-29.

Diez Roux, A., F. Nieto, C. Muntaner, H. Tyroler, G. Comstrock, E. Shahar and et al (1997). "Neighbourhood environments and coronary heart disease: a multilevel analysis." American Journal of Epidemiology **146**: 48-63.

Ellen, I., T. Mijanovich and K. Dillman (2001). "Neighbourhood effects on health: exploring the links and assessing the evidence." Journal of Urban Affairs **23**(3-4): 391-408.

Evans, R. and G. Stoddart (1990). "Producing health, consuming health care." Social Science & Medicine **31**: 1347.

Green, L. and J. Ottoson (1999). Community and population health, 8th Edition. Boston, McGraw-Hill.

Greenland, S., M. Gago-Dominguez and J. Castelao (2004). "The Value of Risk-Factor ("Black-Box") Epidemiology." Epidemiology **15**(5): 529-535.

Haack, S. (2004). "An Epistemologist Among the Epidemiologists." Epidemiology **15**(5): 521-523.

Heller, K., R. Price, S. Reinharz, S. Riger and A. Wandersman (1984). Psychology and community change: Challenges of the future. Homewood, IL, Dorsey.

Krieger, N. (1994). "Epidemiology and the web of causation: has anyone seen the spider?" Social Science & Medicine **39**(7): 887-903.

Krieger, N. (2000). Discrimination and Health. Social Epidemiology. L. Berkman and I. Kawachi. New York, Oxford University Press.

Krieger, N. (2001). "Theories for social epidemiology in the 21st century: an ecosocial perspective." International Journal of Epidemiology **30**: 668-677.

Macintyre, S. and A. Ellaway (2003). Neighborhoods and Health: An Overview. Neighborhoods and Health. I. Kawachi and L. Berkman. Oxford, Oxford University Press.

Macintyre, S., S. Maciver and A. Sooman (1993). "Area, class, and health: should we be focusing on places or people?" Journal of Social Policy **22**: 213-234.

Matthews, S. and M. Meaney (2005). Maternal Adversity, Vulnerability and Disease. Perinatal Stress, Mood and Anxiety Disorders. From Bench to Bedside. A. Riecher-Rossler and M. Steiner. Basel, Karger.

Mayo, D. and A. Spanos (2004). "When Can Risk-Factor Epidemiology Provide Reliable Tests." Epidemiology **15**(5): 523-524.

Misra, D., B. Guyer and A. Allston (2003). "Integrated perinatal health framework. A multiple determinants model with a life span approach." American Journal of Preventive Medicine **25**(1): 65-75.

Morenoff, J. (2003). "Neighbourhood mechanisms and the spatial dynamics of birth weight." AJS **108**(5): 976-1017.

Pearce, N. (1996). "Traditional epidemiology, modern epidemiology, and public health." American Journal of Public Health **86**: 678-683.

Robert, S. (1999). "Socioeconomic position and health: the independent contribution of community socioeconomic context." Annual Review of Sociology **25**: 489-516.

Susser, E. (2004). "Eco-Epidemiology: Thinking Outside the Black Box." Epidemiology **15**(5): 519-520.

Susser, M. and E. Susser (1996). "Choosing a future for epidemiology: II. From black box to Chinese boxes and eco-epidemiology." American Journal of Public Health **86**: 674-677.

Taylor, S. and R. Repetti (1997). "Health psychology: what is an unhealthy environment and how does it get under the skin?" Annual Review of Psychology **48**: 411-447.

Wandersman, A. and P. Florin (2000). Citizen Participation and Community Organizations. Handbook of Community Psychology. J. Rappaport and E. Seidman. New York, Kluwer Academic/Plenum Publishers.

Wandersman, A. and M. Nation (1998). "Urban Neighbourhoods and mental health: Psychological contributions to understanding toxicity, resilience, and interventions." American Psychologist **53**(6): 647-656.

Zimmerman, M. (2000). Empowerment Theory: Psychological, Organisational and Community Levels of Analysis. Handbook of Community Psychology. J. Rappaport and E. Seidman. New York, Kluwer Academic/Plenum Publishers.
